# A toxin-based approach to neuropeptide and peptide hormone discovery

**DOI:** 10.3389/fnmol.2023.1176662

**Published:** 2023-08-31

**Authors:** Thomas Lund Koch, Joshua P. Torres, Robert P. Baskin, Paula Flórez Salcedo, Kevin Chase, Baldomero M. Olivera, Helena Safavi-Hemami

**Affiliations:** ^1^Department of Biomedical Sciences, University of Copenhagen, Copenhagen, Denmark; ^2^Department of Biochemistry, University of Utah, Salt Lake City, UT, United States; ^3^School of Biological Sciences, University of Utah, Salt Lake City, UT, United States; ^4^The Ohio State University College of Medicine, Columbus, OH, United States; ^5^Department of Neurobiology, University of Utah, Salt Lake City, UT, United States

**Keywords:** toxins, venom, neuropeptide, hormone, *Conus*, crustacean hyperglycemic hormone

## Abstract

Peptide hormones and neuropeptides form a diverse class of bioactive secreted molecules that control essential processes in animals. Despite breakthroughs in peptide discovery, many signaling peptides remain undiscovered. Recently, we demonstrated the use of somatostatin-mimicking toxins from cone snails to identify the invertebrate ortholog of somatostatin. Here, we show that this toxin-based approach can be systematically applied to discover other unknown secretory peptides that are likely to have signaling function. Using large sequencing datasets, we searched for homologies between cone snail toxins and secreted proteins from the snails’ prey. We identified and confirmed expression of five toxin families that share strong similarities with unknown secretory peptides from mollusks and annelids and in one case also from ecdysozoans. Based on several lines of evidence we propose that these peptides likely act as signaling peptides that serve important physiological functions. Indeed, we confirmed that one of the identified peptides belongs to the family of crustacean hyperglycemic hormone, a peptide not previously observed in Spiralia. We propose that this discovery pipeline can be broadly applied to other systems in which one organism has evolved molecules to manipulate the physiology of another.

## 1. Introduction

Neuropeptides and peptide hormones (collectively referred to as signaling peptides) are important signaling molecules found throughout the Animal Kingdom (Jékely, 2013; Nikitin, 2015; Koch and Grimmelikhuijzen, 2019), controlling and regulating many diverse biological functions, ranging from metabolism and hunger to learning, pain, and mating (Russo, 2017). Evolutionarily, many signaling peptides are ancient with origins that can be traced back to the common bilaterian ancestor (Jékely, 2013; Mirabeau and Joly, 2013). These peptides typically bind to and activate conserved G protein-coupled receptors (GPCRs) (Mirabeau and Joly, 2013; Vaudry and Seong, 2014).

Signaling peptides are with few exceptions 5–50 amino acids in length and released from larger precursors by specific proteases that cleave at basic or dibasic amino acid residues. The precursors contain an N-terminal signal sequence that targets the protein to the secretory pathway and typically also contain spacer regions of mostly unknown functions followed by the peptide region. This complex precursor structure is accompanied by contrasting patterns of evolution. The signal sequence and spacer regions often diverge substantially between orthologs, whereas the peptide region and flanking proteolytic processing sites are conserved (Williams et al., 2000; Toporik et al., 2014; Foster et al., 2019; Koch et al., 2022). Thus, comparative sequence analysis of the precursors tends to show a pattern of close to neutral selection in the signal sequence and spacer regions, and purifying selection in the region encoding the peptide.

Given their importance in animal biology, extensive research programs have attempted to discover and describe signaling peptides and their receptors. Currently, several hundred different signaling peptides are recognized in humans (Secher et al., 2016; Foster et al., 2019; Tai et al., 2020). Still, the endogenous ligands for almost 100 human GPCRs remain unknown, suggesting additional signaling peptides remain to be identified (Laschet et al., 2018). However, *de novo* discovery of signaling peptides is difficult. Many of the yet unknown signaling peptides may not be highly expressed, may be unstable, or may only be expressed in specific cell types or developmental stages. Bioinformatic approaches to discovery have proven successful in some cases (Fukusumi et al., 2003; Sonmez et al., 2009), but are typically limited to identifying homologs of known signaling peptides from related species. Furthermore, the emerging field of microproteins has also demonstrated that the space of translated small proteins is much larger than previously recognized, even in vertebrate model systems (Saghatelian and Couso, 2015; Ruiz-Orera and Albà, 2019; Hu et al., 2021). These observations call the comprehensiveness of earlier mining efforts into question. True *de novo* computational discovery tends to have a high rate of false positives and any additional evidence for distinguishing true signaling peptides from falsely predicted ones is highly valuable.

We and others have previously shown that some venomous animals have evolved toxins that specifically mimic the signaling peptides of their prey or predators (Cruz et al., 1987; Safavi-Hemami et al., 2016; Sachkova et al., 2020; Eagles et al., 2022). We refer to these as “doppelganger toxins.” Most doppelganger toxins have originated from an endogenous signaling peptide gene that, following recruitment into the venom gland, experienced positive selection to ultimately mimic the related peptide of the target organism (Safavi-Hemami et al., 2016; Sachkova et al., 2020; Koch et al., 2022). This process can be accompanied by the generation of novel, advantageous features of the toxin compared to the endogenous peptide it evolved from, such as enhanced stability, receptor subtype selectivity, or faster action (Safavi-Hemami et al., 2016; Xiong et al., 2020; Ramiro et al., 2022).

Since doppelganger toxins typically share sequence similarity with the signaling peptide they mimic, it is possible to identify these toxins through homology searches. This has for example led to the discovery of con-insulins; weaponized insulins derived from the endogenous cone snail insulin that mimic the insulin expressed in prey (Safavi-Hemami et al., 2016), and the arachnid toxin Ta1a, which has common ancestry with crustacean hyperglycemic hormone (Undheim et al., 2015). While this approach has identified toxins homologous to known signaling peptides, it can also, in principle, be “reversed” i.e., to use toxins as queries to find yet unknown signaling peptides.

Anecdotal evidence has shown that this is possible. Bombesin, a peptide from the poisonous secretions of the European fire-bellied frog (*Bombina bombina*) that stimulates the release of gastrin led to the discovery of homologous peptides in vertebrates (gastrin-releasing peptide (GRP) and neuromedins) (McDonald et al., 1979; Minamino et al., 1983). Similarly, the sea anemone toxin ShK-like1 was used to discover the previously unknown signaling peptide Shk-like2 in the nervous system of cnidarians (Sachkova et al., 2020). Additionally, we recently showed that somatostatin-like toxins from cone snails revealed the presence of a somatostatin signaling system in protostomes (Koch et al., 2022). Here, we hypothesized that this anecdotally reported, toxin-based approach can be used to systematically unravel the existence of unrecognized signaling peptides.

Cone snails and their toxins represent an ideal system for testing the broader feasibility of this approach. *Conus* is a diverse lineage of ~850 species of venomous marine gastropods (MolluscaBase, 2023) with a large repertoire of hyper-diverse conotoxins. Additionally, cone snails have well-described diets ranging from fish to mollusks and annelid worms (Duda et al., 2001; Puillandre et al., 2014; Olivera et al., 2015). This provides a large library of toxins that evolved to specifically target animals belonging to different phyla.

By performing a systematic search of conotoxins and predicted secreted proteins from cone snail prey, we discover five novel doppelganger toxin families with homology to unknown secretory prey proteins. Based on several lines of evidence, including tissue-specific expression, characteristic evolutionary conservation, and structural similarity, we propose that these proteins most likely encode unrecognized signaling peptides. Our findings serve as a proof of concept for the methodical use of doppelganger toxins for the discovery of unknown signaling peptides. We propose that this approach can be applied to other systems in which one organism has evolved compounds to manipulate the physiology of another. This includes venomous animals and their prey, venomous organisms and their predators, and pathogens and parasites and their hosts.

## 2. Materials and methods

### 2.1. Phylogenetic analyses

COI, 12S, and 16S genes from diverse cone snails and *Californiconus californicus* were downloaded from NCBI. The genes were individually aligned using MAFFT v7.487 and trimmed using trimAl v1.2 to remove all columns with gaps. The tree alignments were subsequently concatenated using FASconCAT-G v1.05. Alignment found in Supplementary Data S1. A maximum likelihood tree was constructed using IQ-TREE v 1.6.12 on a single thread. Based on the Bayesian information criterion the tree was constructed with TVM + F + I + G4 model of evolution. Bootstrap values were calculated with 1,000 replicates using IQ-TREE’s UFBoot method.

### 2.2. Generation of putative signaling peptide databases from prey organisms

The prey databases were built for the fish *Danio rerio*, the mollusk Aplysia californica, and the two annelids *Capitella teleta* and *Platynereis dumerlii*. We chose these species as they are important model organisms of the different *Conus* prey phyla. These organisms are well-studied and have ample sequence material available.

The *Aplysia* database was built using the NCBI Protein database with the query ““*Aplysia californica*” [porgn: __txid6500]” in December 2021 (27,891 sequences). Redundant sequences were removed using cd-hit (−c 0.95) and proteins with signal sequences were extracted using SignalP 6.0 (2,649 sequences). We further added secreted proteins from *A. californica* transcriptomes. Open reading frames encoding proteins with a minimum length of 50 amino acids were extracted with getorf 6.6.0.0 and clustered using cd-hit (−c 0.9). All methionine start-sites were assessed with SignalP6.0 and secreted sequences were retained (for a total of 10,039 sequences). Enzymes were removed from the database with mmseqs at an e-value of 1E-10 to uniport sequence ““Mollusca (9MOLL) [6447]” AND goa: (“catalytic activity [0003824]”)” resulting in a final set of 7,009 secreted proteins.

For the zebrafish database, we downloaded all proteins from the NCBI Protein database with the query ““*Danio rerio*” [porgn:__txid7955]” and altorfs from^1^ based on the Ensembl zebrafish annotation Zv9.97 (total 177,106 sequences). Using the approach above we extracted 5,929 secreted proteins. These were supplemented with 11,562 secreted sequences identified from three assembled transcriptomes. Following removal of sequences with transmembrane domains (11,374 seqs) and similarity to chordate enzymes (Uniprot search terms “taxonomy:“Chordata (9CHOR) [7711]” goa: (“catalytic activity [0003824]”)”) the final zebrafish database consisted of 9,328 sequences.

The annelid database was built from 32,117 sequences downloaded from the NCBI Protein database with the search term: ““Capitella teleta” [porgn:__txid283909],” of which 2,483 sequences had a predicted signal sequence. We also added 11,729 secreted protein sequences identified from four transcriptomes of the annelid *Platynereis dumerlii*. Following removal of transmembrane proteins (12,127 sequences) and enzymes (Uniprot search terms: “taxonomy: “Annelida [6340]” AND goa: (“catalytic activity [3824]”)”) the final annelid database consisted of 10,659 secreted proteins.

Accession numbers of all SRA datasets used in this paper can be found in Supplementary Data S2, accession numbers for TSA or predicted genomic sequences are included in fasta headers. Code is available from.^2^

### 2.3. Venom database preparation

We downloaded 92 transcriptomes from 45 different species of cone snails representing diverse clades with different prey preferences from NCBI (SRA accession numbers listed in Supplementary Data S2). These were assembled as previously described (Koch et al., 2022). The assembled venom gland transcriptomes were processed individually in a process identical to the transcriptome of *A. californica* with slightly different settings (code available from ^2^). The open reading frames were clustered with cd-hit at 100% identity and pooled.

### 2.4. Transcriptome sequencing

We performed additional transcriptome sequencing of *A. californica* and *Conus furvus*. Specimens of *A. californica* were ordered from the National Resource for Aplysia at the University of Miami, FL, United States. Animals were anesthetized as previously described (Zhao et al., 2009). The venom gland of a single specimen of *C. furvus* was also dissected for sequencing. *C. furvus* was included in this study to provide an additional mollusk-hunting cone snail for the analyses. Total RNA was extracted using the Direct-zol RNA extraction kit (Zymo Research), with on-column DNase treatment and an additional wash step after the first purification, according to the manufacturer’s instructions. Library preparation and sequencing were performed by the University of Utah High Throughput Genomics Core Facility as previously described for different cone snail tissues (Koch et al., 2022). The SRA generated in this paper have been deposited with accession numbers SRR22829302, SRR23242094-SRR23242120.

### 2.5. Doppelganger toxin search

The proteins from the prey databases were used to query the combined venom database from cone snail venom gland transcriptomes with blastp. We used a word size 2 and e-value 1e-2 in the searches. A total of 515 sequences from the *Aplysia* database had significant hits, 675 in the zebrafish database, and 1,020 in the annelid database. For each hit in the prey databases, we created a multiple sequence alignment with the venom blast hits with TPM above 10. The alignments were then visually inspected to identify presence of cleavage sites. Alignments that showed a characteristic doppelganger toxin pattern (a combination of highly conserved and diverse amino acid residues in a potential mature peptide region) and putative processing sites were further analyzed by searching for orthologs in closely related species in accordance with the criteria stated in the results.

### 2.6. Evolutionary analysis

Sequences were aligned using MAFFT v7.487 and the evolutionary rates were calculated using rate4site. The evolutionary rates were plotted using a sliding window of 5 amino acids. The boxplots were built from the evolutionary rates of the peptide and pro-peptide regions as shown in the alignment figures (the likely processing sites were left out of the analysis) and compared using Wilcoxon rank-sum test. Rate4site scores have been shown to be strongly correlated with and directly comparable to dN/dS values (Sydykova and Wilke, 2017).

We identified the location, size, and phases of introns using the online version of Splign. The mRNA was obtained from the respective transcriptomes, and the corresponding genomic segment was identified using blastn with standard setting.

Clustering analysis was performed using CLANS (Frickey and Lupas, 2004), which randomly initializes the individual sequences as nodes and performs an all-against-all blastp. The negative logarithm of the blast *p*-values is transformed into an attractive force in addition to a uniform repulsive force between the nodes. We used the BLOSUM62 scoring matrix using the web tool.^3^ The clustering was initially done in 3D and collapsed to 2D for >300,000 rounds, at which point the clustering had converged.

### 2.7. Structural prediction and comparison

We obtained structural predictions of the toxins and putative signaling peptides using a combination of AlphaFold2 neural network and MMSeqs2 to obtain a multiple sequence alignment. These are combined in ColabFold, where the full precursor sequences were used as the query sequence. The best of five Amber relaxed models was selected. We used the protein structural comparison server DALI to compare the predicted toxin and signaling peptide structures to all available protein structures in PDB and different species subsets of the AlphaFold database.

### 2.8. Protein extraction and mass spectrometry

Dissected venom glands were homogenized using Teflon pestles in 200 μL 40% acetonitrile (ACN), 0.1% trifluoroacetic acid (TFA). Following centrifugation, supernatants were diluted 1:4 in water. The pH was adjusted to 8 in 100 mM triethylammonium bicarbonate and an aliquot of the sample was reduced using 40 mM dithiothreitol for 60 min at 60°C. An aliquot of this sample was further alkylated using 40 mM iodoacetamide for 30 min in the dark and digested with trypsin at 0.2 μg/mL overnight at 37°C. The peptides were resuspended in 300 μL of 0.1% TFA and desalted using Pierce™ Peptide Desalting Spin Columns according to the manufacturer’s instructions. The peptides were resuspended in 50 μL of 0.1% formic acid and further diluted 1:5 in 0.1% formic acid for LC–MS/MS analysis. Reversed-phase nano-LC–MS/MS was performed on an UltiMate 3,000 RSLCnano system (Dionex) coupled to a Thermo Scientific Q Exactive-HF mass spectrometer equipped with a nanoelectrospray source. 2 μL of each sample were first trapped on a 2 cm Acclaim PepMap-100 column (Thermo Scientific) with 5% acetonitrile at 5 μL/min and at 5 min the sample was injected onto the liquid chromatograph reverse-phase Acclaim™ PepMap™ 100 C18 2.0 μm nanocolumn (Thermo Scientific). A 500 mm long/ 0.075 mm inner diameter nanocolumn heated to 35°C was employed for chromatographic separation. The peptides were eluted with a gradient of reversed-phase buffers (Buffer A: 0.1% formic acid in 100% water; Buffer B: 0.1% formic acid in 100% acetonitrile) at a flow rate of 0.2 μL/min. The LC run lasted for 85 min with a starting concentration of 5% buffer B increasing to 28% buffer B over 75 min, up to 40% buffer B over 10 min and held at 90% B for 10 min. The column is allowed to equilibrate at 5% buffer B for 20 min before starting the next data acquisition. The mass spectrometer was operated in data-dependent acquisition MS/MS analysis mode selecting the top 20 most abundant precursor ions between 375–1,650 m/z at 60,000 resolution for fragmentation at 15,000 resolution. Data were analyzed using Xcalibur software (Thermo Scientific) and Byonic (Protein Metrics). The raw files have been deposited at PRIDE with accession number PXD038986, PXD038992, PXD038993.

## 3. Results

### 3.1. Discovery of five novel doppelganger toxins and homologous secreted prey peptides

We aimed to identify novel prey signaling peptides that share sequence similarities to conotoxins in model organisms from the three phyla of cone snail prey: the chordate *Danio rerio*, the mollusk *Aplysia californica*, and the two annelids *Capitella teleta* and *Platynereis dumerilii*. To this end, we constructed libraries of secreted proteins from prey species, which, in principle, should contain all known and unknown signaling peptides (Supplementary Figure S1). This provided us with a set of 9,328 unique sequences from *D. rerio*, 7,009 sequences from *A. californica*, and 10,659 sequences from the two annelids *C. teleta* and *P. dumerilii*. In addition, we built a database of secreted proteins from 92 venom gland transcriptomes of 45 cone snail species. These cone snails belong to 20 phylogenetically diverse clades with different prey preferences (Supplementary File S2), resulting in a library of 25,989 sequences of conotoxins and conotoxin candidates, principally containing all conotoxins.

We employed the following criteria to identify putative new signaling peptides: (I) The protein must be predicted to be secreted. (II) The prey protein must yield at least two homology hits (*e*-value >0.01) to the cone snail database. (III) The prey protein must either have classical signaling peptide processing sites or the peptide must span the entire precursor except for the signal sequence. (IV) There must be orthologs in closely related organisms that also show the characteristics of signaling peptide precursors. (V) Neither the prey protein nor its orthologs should already have a functional annotation. Using these criteria, we identified five families of secreted proteins from mollusks and annelids that potentially encode novel signaling peptides. We refer to these as doppelganger-related peptides (DREPs). DREPs were named based on their sequence or structural characteristics.

#### 3.1.1. Triangle DREPs

**Figure 1:**
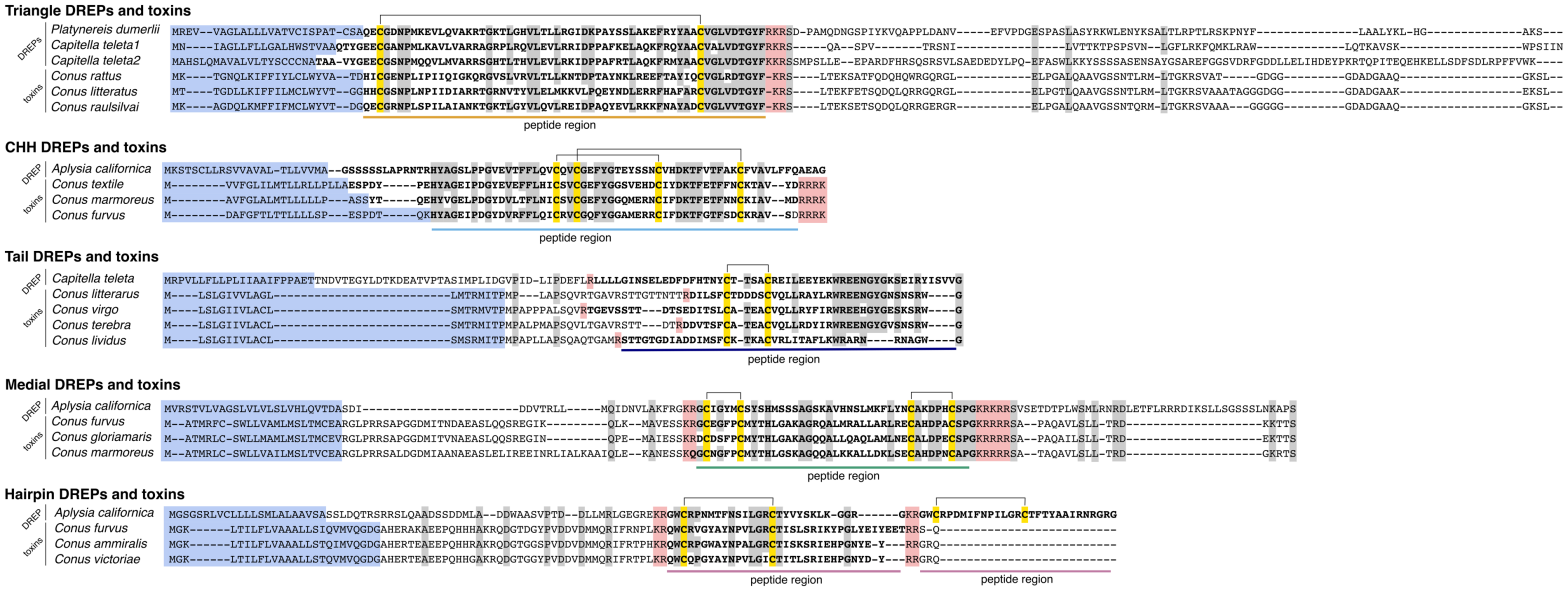
Multiple sequence alignments showing high similarity of the five identified doppelganger-related peptides (DREPs) families with doppelganger toxin precursors. Signal sequences are highlighted in blue; cysteines are in yellow with disulfide bonds shown as connecting lines, and processing sites are highlighted in red. Mature DREP and toxin regions are in bold and underlined. Identical amino acids are highlighted in gray.

The first family of putative signaling peptides were discovered from a *P. dumerilii* transcript (GenBank ID: HAMN01029001) and two sequences from *C. teleta* (ELT98797, ELT98795) with multiple hits in the toxin dataset (Figure 1). The predicted DREPs and toxins are 59–65 amino acids long, contain 10.3% acidic and 13.7% basic residues and a single disulfide bond formed by two cysteines. We identified 29 related toxin gene sequences from 11 different cone snail species belonging to the *Africonus*, *Elisaconus*, and *Rhizoconus* worm-hunting clades (Supplementary Figure S2 and Supplementary Data S3).

#### 3.1.2. CHH DREPs

The second DREP family was discovered from hits to a transcript from *A. californica* (GBCZ01041960) (Figure 1). The predicted peptide is 71 amino acids in length, contains two disulfide bonds (16.3% acidic, 7.3% basic), and is located immediately downstream of the signal sequence. We identified homologous toxin sequences from the snail-hunting *Conus textile*, *Conus marmoreus*, and *Conus furvus* (Supplementary Figure S2 and Supplementary Data S4), suggesting that the venom recruitment event happened once in the common ancestor of snail hunters.

#### 3.1.3. Tail DREPs

The third DREP family was discovered from hits to a predicted *C. teleta* protein (ELT87057) (Figure 1). The peptide is 53 amino acids long, located in the C-terminus of the precursor, and contains a single N-terminal disulfide bond (12.1% acidic, 9.6% basic). We identified eight conotoxins with sequence similarity from species belonging to the worm-hunting clades of *Africonus*, *Elisaconus*, *Lividoconus*, and *Virgiconus* (Supplementary Figure S2 and Supplementary Data S5).

#### 3.1.4. Medial DREPs

This family was identified from an *A. californica* sequence (XP_005095677) (Figure 1). The predicted peptide region is in the medial region of the precursor, 40 amino acids in length (8.5% acidic, 6.7% basic), and predicted to contain a C-terminal amide and two disulfide bonds. We note that this family of DREPs may encode two peptides rather than a single peptide spanning the entire region (further discussed below). However, canonical processing sites for this cleavage are only present in some precursors. We identified 13 toxin sequences in the venom gland transcriptomes; all from snail-hunting species of the *Calibanus*, *Conus*, and *Cylinder* clades (Supplementary Figure S2 and Supplementary Data S6).

#### 3.1.5. Hairpin DREPs

The final DREP family was identified from hits to a protein from *A. californica* (XP_005089801) (Figure 1). This protein has previously been suggested to encode a signaling peptide based on similarity to a toxin derived from the cone snail *Conus victoriae* from the *Cylinder* clade, contryphan-Vc1 (Robinson et al., 2016). Here, we identified similar toxins in *C. furvus* and *Conus ammiralis*, two additional snail-hunting species (Supplementary Figure S2 and Supplementary Data S7). The toxins contain 7.2% acidic and 9.3% basic amino acid residues. As previously observed, the signal sequence of these toxins is similar to that of the contryphans/O2 superfamily of conotoxins (Robinson et al., 2014). However, apart from the signal sequence the doppelganger toxin family shares limited similarity with contryphans. The unusual evolution of this doppelganger toxin family will be addressed in more detail below.

We note that homology searching identified unknown signaling peptides in both mollusks and annelids but did not detect any novel signaling peptides in zebrafish, possible due to the phylogenetic distance between predator and prey.

### 3.2. Doppelganger-related peptides are widely present in mollusks and annelids, including cone snails

**Figure 2:**
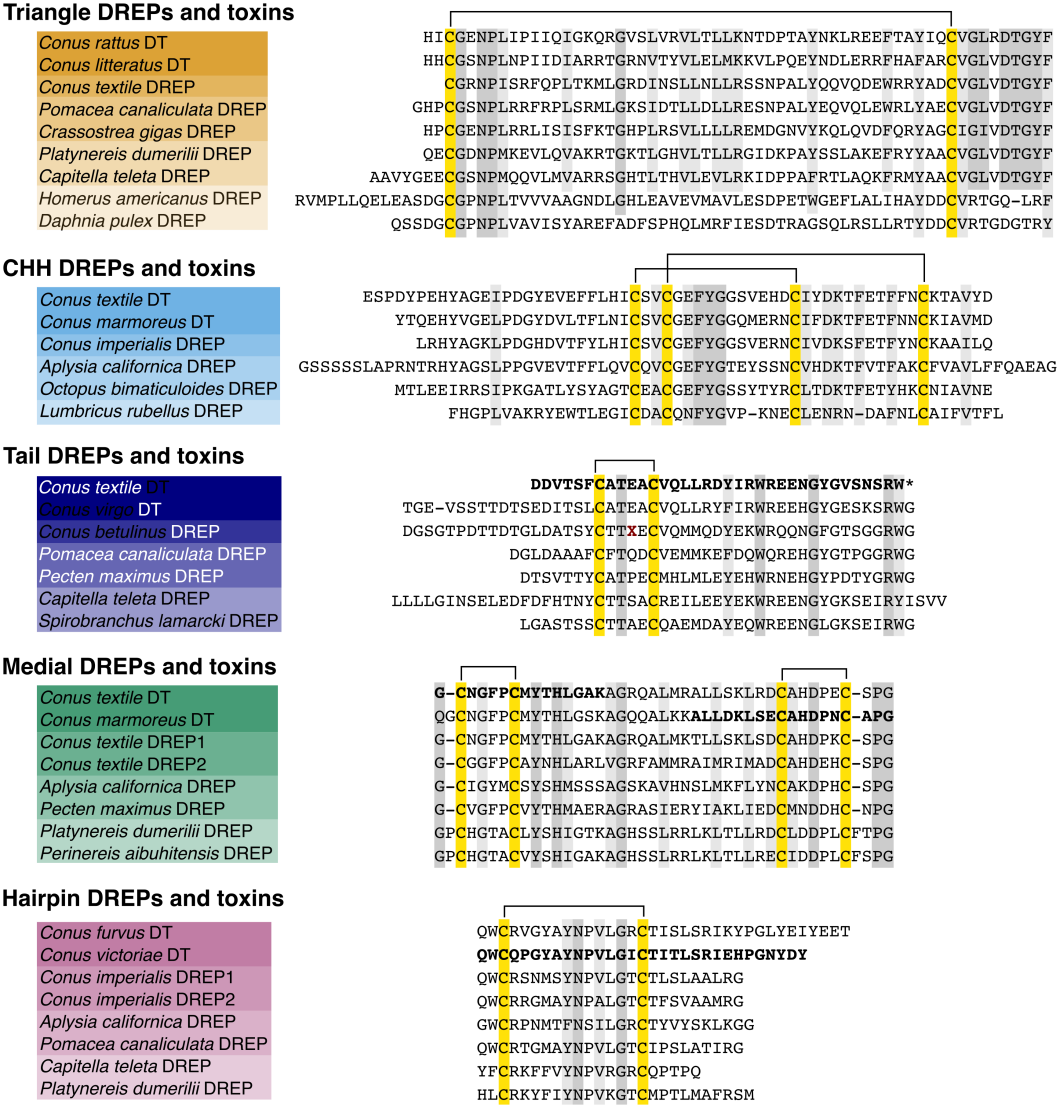
Multiple sequence alignment of representative mature toxins and signaling peptides of the five doppelganger toxin (DT) and DREP families. Alignments highlight high sequence similarity of the toxins and DREPs, including conserved cysteine scaffolds. Two endogenous Medial and Hairpin DREPs are found in cone snails (DREP1 and 2). The *Conus betulinus* Tail DREP is only a partial sequence with a sequencing error (dark red X). Bold sequences were detected using tandem mass spectrometry sequencing of extracted venom.*: amidation.

Having established the presence of DREPs in cone snail prey, we searched for orthologs in other organisms and could identify all five families in other mollusks and annelids (Figure 2 and Supplementary Data 8). The precursors are all secreted, have similar precursor architecture, identical number of cysteines, and similar processing sites as the initial prey sequences. Using psi-blast, we further identified genes encoding Triangle DREP-like peptides in additional protostome phyla, including Arthropoda and Platyhelminthes with identical structure (Figure 2 and Supplementary Data S9). Clustering analyses further show homology of the larger Triangle DREP family (Supplementary Figure S3). Lastly, we found that each of the five DREP families shares common intron position and phase across phyla (Supplementary Data S10), corroborating that the identified protostome sequences are homologous.

To investigate if the doppelganger toxins identified originated from endogenous cone snail signaling peptides, we queried *Conus* circumoesophageal nerve ring transcriptomes for sequences that could have given rise to the doppelganger toxins. In all but one case (Tail DREP) we could recover homologous transcripts in nerve rings of *C. textile* and *Conus rolani* (Figure 2 and Supplementary Data S11). Multiple sequence alignment of the nerve ring precursors with the corresponding toxins clearly shows homology (Figure 2), even into the 3′ and 5′ untranslated regions (Supplementary Data S12). They also show the same features of intron positions and phases identified above (Supplementary Data S10), demonstrating that the doppelganger toxins evolved from the conserved *Conus* nerve ring DREPs.

### 3.3. Doppelganger toxins are highly expressed in venom glands

Conotoxin expression typically ranges from 10–100,000 transcripts per million (TPM) (Phuong et al., 2016; Robinson et al., 2017). While some low-expression transcripts from the venom gland encode non-toxin proteins, highly expressed transcripts almost certainly encode conotoxins. When we quantified the expression of the five doppelganger toxins, we found at least one highly expressed transcript (> 1,000 TPM) in each of the families, supporting that these are indeed toxins that are functionally important in some cone snail species (Supplementary Figure S4).

To determine if the doppelganger toxins are processed into mature peptides, we performed high-resolution tandem mass spectrometry (MS/MS) of venom extracted from cone snails with high transcription of the toxins. While we could not detect Triangle or CHH doppelganger toxins, we identified the 35-residue Tail doppelganger toxin as an [M + 3H]^+3^ ion of *m/z* 828.396 (calculated *m/z* = 828.392) in the reduced venom extract of *Conus terebra* (Supplementary Figure S5A). Tandem MS/MS sequencing confirmed the predicted sequence of the toxin in this venom (Supplementary Figure S5A). We further identified two separate peptides from the precursors of the Medial doppelganger toxins from the extracted venoms of *C. textile* and *C. marmoreus*. The 15-residue *C. textile* toxin representing the N-terminally located peptide was identified as an [M + 3H]^+3^ ion of *m/z* 800.342 (calculated *m/z* = 800.341) in reduced and alkylated venom extract (Supplementary Figure S5B). The C-terminally located peptide was identified in the reduced and alkylated venom extract of *C. marmoreus*. It consists of 19 residues and has a monoisotopic [M + 2H]^+2^ ion of *m/z* 984.450 (calculated *m/z* = 984.451) (Supplementary Figure S5C). While not identified here, the peptide contryphan-Vc1, a member of the Hairpin doppelganger toxins, has previously been identified in the venom of *C. victoriae* using MS/MS (Robinson et al., 2016), further confirming that the doppelganger toxins are processed into mature venom peptides.

### 3.4. Tissue-specific transcriptomes demonstrate expression of DREPs in neuroendocrine and secretory tissues

If the identified DREPs encode signaling peptides, we hypothesize that these are expressed in neuroendocrine tissues. To test this, we quantified DREP expression in tissue-specific transcriptomes of *A. californica* (generated here) and publicly available datasets of the mollusk *Doryteuthis pealeii* (longfin inshore squid) and annelid *Lumbricus rubellus* (red earthworm).

*Aplysia* CHH DREP is expressed in eight sequenced ganglia and nerves and absent in non-neuronal transcriptomes (Supplementary Figure S6A). *Aplysia* Medial DREP is exclusively expressed in the pleural ganglion, albeit at low levels (9.23 TPM). *Aplysia* Hairpin DREP is expressed in all neuronal transcriptomes but is highly expressed in the salivary gland and has low expression in the foot. Triangle and Tail DREPs were seemingly absent in the *Aplysia* dataset. In the *D. pealeii* transcriptomes, Triangle DREP is highly expressed in neuronal tissues, and to a much lower degree in the testes and buccal mass. CHH DREP is expressed in the brain and brachial lobe, and Hairpin DREP expression was detected in neuronal tissues, but also in some non-neuronal secretory tissues (Supplementary Figure 6B). We further observed expression of Triangle and Hairpin DREPs in the nerve cord and neural ganglion of *L. rubellus*, while CHH DREP is expressed in the body wall and the clitellum, and Tail DREP in the calciferous and digestive tissue (Supplementary Figure S6C).

Our combined findings from *A. californica, D. pealeii*, and *L. rubellus* show that the DREPs are encoding peptides expressed primarily in neuroendocrine and secretory tissues.

### 3.5. Doppelganger toxins and DREPs only show similarity in the peptide regions

While doppelganger toxins evolved to mimic the signaling peptides of their target organism, the non-toxin-encoding regions of the precursors are presumably under little if any evolutionary pressure to mimic the signal sequence or the pro-region(s) of the prey precursor. Thus, we hypothesize that the precursors of doppelganger toxins and their DREPs may only show significant similarity in the region that encodes the mature peptide. To investigate this, we aligned each of the toxins to their respective DREPs and quantified the number of identical amino acids in the signal sequence, the peptide region, and the spacer region(s). Indeed, we found that the toxins only show significant similarity to the prey protein in the peptide region (Supplementary Figure S7). Due to the low number of toxins for Hairpin DREPs (*n* = 3), we were not able to statistically quantify the amino acid percentage identity in the different regions. However, in the other four cases, there is a clear trend toward higher similarity in the peptide region. Overall, we find that this region displays between 35–55% identity compared to only 12–28% for the signal sequences and spacer regions.

### 3.6. Evolutionary trace analyses show contrasting patterns of conservation in doppelganger toxins and DREPs

We have previously shown that signaling peptides and conotoxins evolve under contrasting selection pressures (Koch et al., 2022). Whereas signaling peptide precursors show a high level of conservation in the mature peptide region compared to the signal and spacer regions, toxin precursors have conserved signal sequence and spacer regions but are extremely diverse in the toxin region (Woodward et al., 1990; Fry et al., 2009). To investigate if this pattern of evolution is also present in the identified doppelganger toxins and DREPs, we performed evolutionary trace analyses.

**Figure 3:**
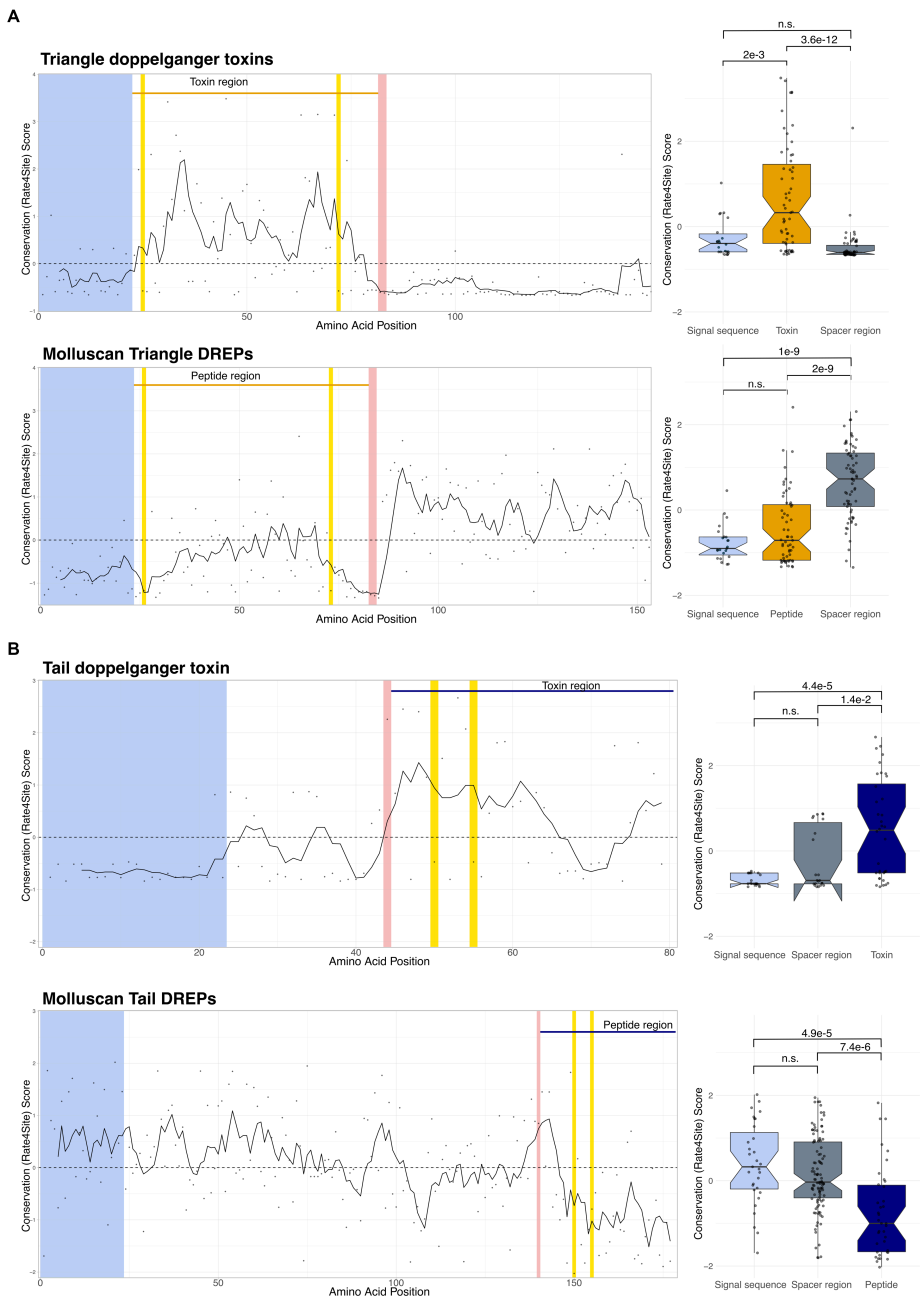
Evolutionary trace analyses show different conservation (rate4site) scores in the toxin/peptide regions compared to the signal sequence and spacer region(s). Position-specific rate4site scores for Triangle doppelganger toxin represented by *Conus litteratus* doppelganger toxin (DT) and molluscan Triangle DREPs represented by the endogenous *Conus textile* precursor. Wilcoxon rank-sum test shows significant differences between the toxin region compared to the signal sequence and spacer region. The signal peptide is depicted in light blue, processing sites are in red, and cysteines in yellow. The peptide and toxin regions are shown above the graphs. Spacer regions are defined as the non-signal sequence/peptide/processing site regions. Additional traces can be found in Supplementary Figure S8.

Despite differences in the evolutionary trace analyses for the five doppelganger toxins and DREP families, we observe contrasting patterns of evolution between the toxin and signaling peptide sequences (Figure 3 and Supplementary Figure S8). The toxin regions are, on average, more divergent than the surrounding spacer regions and signal sequence. In contrast, the DREP precursors are generally well-conserved in the peptide-encoding region. This is consistent with diversification of toxin genes following recruitment from a conserved endogenous signaling gene into the venom gland. Thus, the doppelganger toxins and DREPs show the characteristic pattern of evolutionary conservation predicted for doppelganger toxins and signaling peptides.

### 3.7. Structural predictions show identical structures despite limited sequence similarity

Signaling peptide action is mediated via binding to membrane proteins and is contingent on the complementarity of the receptor ligand-binding site and the tertiary structure of the peptide ligand. We hypothesized that doppelganger toxins conserve their three-dimensional structure through evolution to preserve the ability to modulate the prey receptor. To investigate this, we obtained structural predictions of doppelganger toxins and their prey DREPs using AlphaFold2 (Jumper et al., 2021).

**Figure 4:**
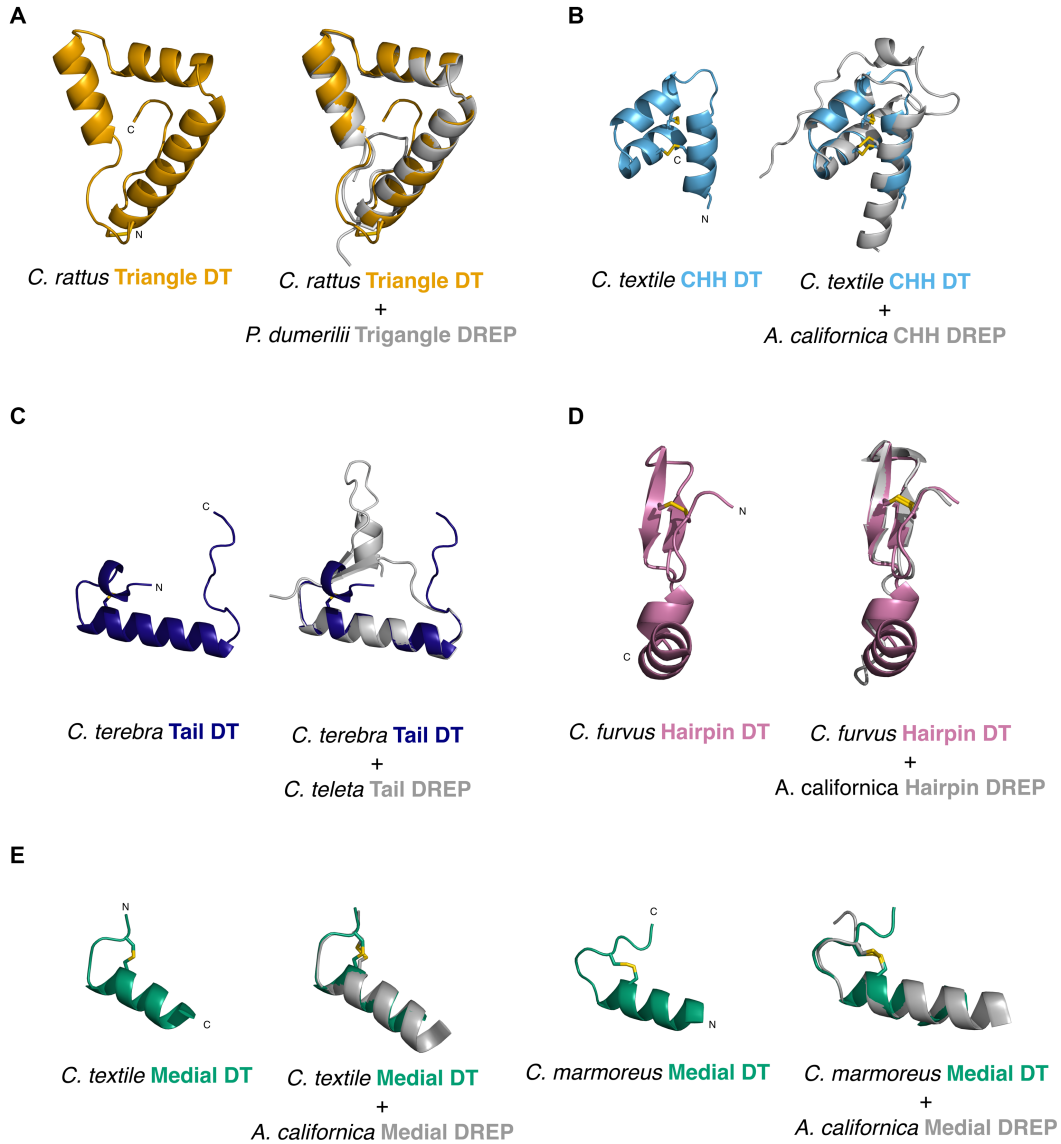
Structural predictions (Alphafold2) and alignments of doppelganger toxins (DT) and doppelganger-related peptides (DREPs) highlight their structural similarity despite limited sequence identity. **(A)** *Conus rattus* Triangle DT 1 (left) and alignment with *Platynereis dumerilii* Triangle DREP (right). **(B)** *Conus textile* CHH DT (left) and alignment with *Aplysia californica* CHH DREP (right). **(C)** *Conus terebra* Tail DT (left) and alignment with *Capitella teleta* Tail DREP (right). **(D)** *C. furvus* Hairpin DT (left) and alignment with *A. californica* Hairpin DREP (right). **(E)** *Conus textile* and *marmoreus* Medial DTs (top) and respective alignment with *A. californica* Medial DREP (bottom).

For the Triangle DREP, both the *Conus rattus* toxin and *P. dumerilii* DREP predicted structures form three alpha-helices in a triangular loop linked by a disulfide bond (average pLDDT 72.4 and 67.6, respectively) (Figure 4). Even though the sequences have <50% identity, the predicted structures overlap very well (rmsd 0.71).

Structural predictions of the *Aplysia* CHH DREP and *C. textile* toxin both have three alpha-helices connected by disulfide loops with an additional short helix in the flexible N-terminus of the *Aplysia* peptide (average pLDDT 91.7 and 67.0). Structural alignment has rmsd of 1.1.

The predicted structure of the Tail doppelganger toxin from *Conus terebra* (average pLDDT 69.1) has a single alpha-helical region located C-terminally of the single cysteine loop (Figure 4). The matching Tail DREP structure from the annelid *C. teleta* (average pLDDT = 67.14) also shows a single alpha-helical region following the cysteine loop and contains a short segment of parallel beta-sheets toward the N- and C-termini (rmsd 0.7).

Predictions of the structures of the Hairpin doppelganger toxin from *C. furvus* and Hairpin DREP from *Aplysia* yielded average pLDDT of 70.86 and 55.1, respectively (using the first peptide copy of the Aplysia precursor). While the confidence for the *Aplysia* structure is low, the two peptides align well (1.01 rmsd) (Figure 4). The structure of contryphan-Vc1 from the Hairpin doppelganger family has been experimentally determined (PDB:2 N24) (Robinson et al., 2016) and conforms with the predicted structures (1.83 rmsd to *C. furvus* toxin).

Collectively, the high similarity between the predicted structures of DREPs and their corresponding doppelganger toxins despite a low sequence similarity further suggests that the toxins specifically mimic the identified DREPs.

### 3.8. Structural predictions of CHH DREP identify the first spiralian member of the CHH hormone family

Protein three-dimensional structures are more conserved than the corresponding amino acid sequences (Illergård et al., 2009). We therefore tested if any of the predicted DREP structures showed resemblances to known peptides with experimentally verified structures by using the structural similarity search DALI (Holm, 2022).

**Figure 5:**
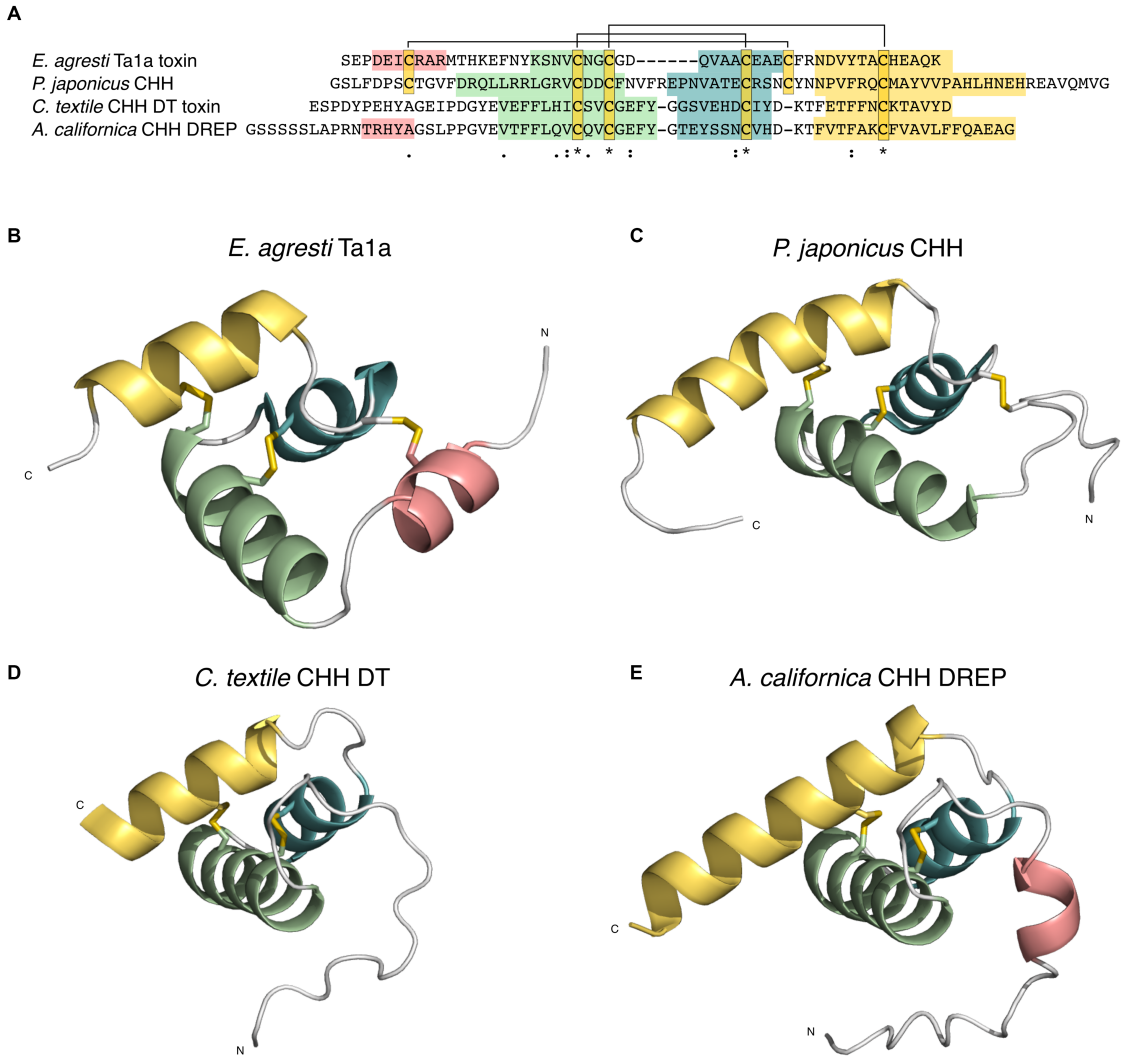
Structural similarity suggests that the CHH doppelganger-related peptides (DREP) family is related to the crustacean hyperglycemic hormone (CHH)-superfamily of signaling peptides. **(A)** Multiple sequence alignment of funnel spider (*Eratigena agrestic*) Ta1a CHH-toxin, *Panaeus japonicus* CHH, *Conus textile* CHH doppelganger toxin (DT), and *Aplysia californica* CHH DREP show limited sequence similarity and share only two out of three disulfide loops. Coloration corresponds to alpha helices shown in B-E. **(B)** Ta1a toxin from funnel spider *E. agresti* (PDB: 2KSL), **(C)** *P. japonicus* CHH (PDB: 5B5I), **(D)** Alphafold2 structural prediction of *C. textile* CHH DT, **(E)** Alphafold2 structural prediction of *A. californica* CHH DREP. **(B–D)** have similar tertiary structures.

Whereas most searches did not retrieve any significant hits, a search for structural homologs of the *Aplysia* CHH DREP yielded several close matches. The top hits were k-Ssm1a (PDB: 2 M35) and Ssd609 (PDB: 2MVT) toxins from the centipede *Scolopendra subspinipides*, the insecticidal toxin Ta1a from the funnel spider *Eratigena agrestic* (PDB: 2KSL), and crustacean hyperglycemic hormone (CHH) (PDB: 5B5I) of the kuruma prawn, *Panaeus japonicus*. There is high structural resemblance between the predicted structure of the *Aplysia* peptide to both the k-Ssm1a and Ssd609 toxins (2.1 and 1.9 rmsd) and the kuruma prawn CHH (2.7 rmsd), even though the sequences only share 26%, 19%, and 14% sequence identity, respectively (Figure 5). The structural similarity strongly suggests that CHH DREPs encode signaling peptides belonging to the CHH superfamily. Furthermore, with two exons separated by a phase 2 intron, the CHH DREP mirrors the proposed gene structure of the ancestral ecdysozoan CHH gene (Montagné et al., 2010), supporting that the CHH DREPs belong to the CHH superfamily. This is the first example of a signaling peptide belonging to the CHH family found outside of Ecdysozoa and confirms that the method employed here indeed can be used to discovery unknown signaling peptides in the cone snail prey.

### 3.9. Hairpin toxins evolved through exon shuffling

**Figure 6:**
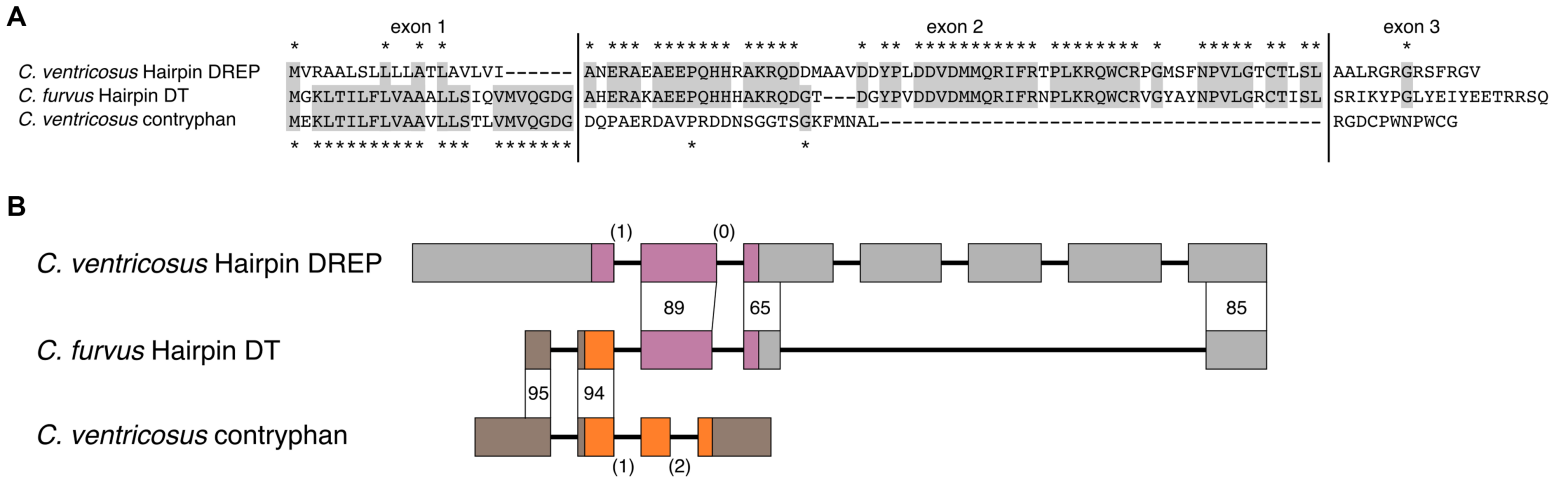
Hairpin doppelganger toxins evolved by exon shuffling of the cone snail endogenous Hairpin DREP and contryphans. **(A)** Multiple sequence alignment of the amino acid sequences shows high similarity of Hairpin doppelganger toxin to contryphan/O2 toxin in the signal sequence region located on the first coding exon, and a high similarity to cone snail endogenous Hairpin DREP in the second exon encoding the mature toxin. **(B)** Genes of *Conus ventricosus* Hairpin DREP and contryphan are consistent with an origin of Hairpin doppelganger toxin by exon shuffling. The *C. ventricosus* Hairpin DREP gene consists of 7 exons (wide boxes) with the open reading frame located on exons 1-3 (purple; UTR: gray) separated by a phase 1 and phase 0 intron (shown in parantheses). The *C. ventricosus* contryphan gene consists of 4 exons with the open reading frame located on exons 2-4 (orange) separated by a phase 1 and phase 2 intron. The *C. furvus* doppelganger toxin gene shares high identity with the 3’ UTR region of contryphan exon 1 and exon 2 (95 and 94 %), and high identity with Hairpin DREP exon 2, 5’ end of exon 3, and 5’ end of exon 7 (89, 65, 85 %).

The underlying molecular mechanisms of conotoxin diversity and recruitment is not fully understood. While differential rates of evolution in the distinct functional units of conotoxin precursors play an important role, other mechanisms have also been proposed (Pi et al., 2006). We noticed that the signal sequence of Hairpin doppelganger toxins belongs to the contryphan/O2 toxin superfamily, but the remaining regions are very distinct from other sequences in this superfamily. Furthermore, the Hairpin toxins are highly similar to the endogenous cone snail DREP - but only in the spacer and peptide regions (Figure 6). Based on these observations, we hypothesize that Hairpin toxins evolved by exon shuffling to create a contryphan/O2-Hairpin DREP chimera. Exon shuffling has been observed in other venomous animals (Wang et al., 2016).

To investigate this, we identified the endogenous Hairpin DREP and contryphan/O2 toxin genes in the genome of *C. ventricosus* (Pardos-Blas et al., 2021). The Hairpin DREP gene consists of 7 exons with the open reading frame located on exons 1–3 which are separated by a phase 1 and a phase 0 intron (Figure 6). The *C. ventricosus* contryphan genes consists of 4 exons with the venom precursor located on exons 2–4 separated by a phase 1 and a phase 2 intron (Figure 6). When we aligned the *C. furvus* Hairpin toxin to the *C. ventricosus* contryphan gene, we found that the 5’ UTR and the region encoding the signal sequence align with 95% identity, but that the remaining 3′ end only aligns with 28% identity (Supplementary Data S13). Conversely, when the *C. furvus* Hairpin toxin is aligned to the *C. ventricosus* Hairpin DREP gene, there is 18% identity in the 5’ UTR and region encoding the signal sequence, whereas the remaining 3′ end aligns with 65–89% identity (Supplementary Data S13). The Hairpin doppelganger toxins most likely evolved by shuffling of exons 1 and 2 of a contryphan gene with exons 2, 3, and 7 of the Hairpin DREP gene; a fusion made possible by the two phase-1 introns of both the Hairpin DREP and contryphan genes. Since the contryphan superfamily is found throughout *Conus* (Jimenéz et al., 1996; Massilia et al., 2001; Grant et al., 2004), these toxins are evolutionarily older than Hairpin toxins, which so far have only been identified in snail hunters. This leads us to believe that the Hairpin toxins adopted the contryphan signal sequence rather than the other way round.

## 4. Discussion

Signaling peptides are essential to animal biology, but *de novo* discovery of high likelihood candidates is difficult. While bioinformatic approaches have identified some novel signaling peptides, the false positive rate is very high. In this study, we used a method centered around cone snail toxins to identify high likelihood candidates for unknown signaling peptides. Using this approach, we discovered five novel doppelganger toxins and related prey peptides (DREPs) that potentially encode novel signaling peptides. Using structural similarity, we confirm that one of these is indeed a signaling peptide which is related to the ecdysozoan CHH. While this approach cannot be used to globally identify all signaling peptides, recent research has found that doppelganger toxins are present in many venomous animals (Undheim et al., 2015; Sachkova et al., 2020).

The doppelganger toxins we discovered here define new toxin gene superfamilies. First, all the sequences contain distinct N-terminal signal sequences used to classify toxins into superfamilies. Second, at least one member of each superfamily is highly expressed exclusively in the venom glands. Third, using tandem mass spectrometric (MS/MS) sequencing, we confirmed the presence of three of the five translated doppelganger peptides in venom. Fourth, the toxin sequences display conserved signal and spacer regions combined with hypervariable toxin regions; a well-described feature of conotoxins. Fifth, we recovered the endogenous genes that gave rise to the toxins. Finally, we demonstrate the presence of an emerging characteristic pattern of contrasting evolutionary conservation between doppelganger toxins and the DREPs they originated from. Collectively, these findings leave little doubt that the herein identified doppelganger toxins are *de facto* conotoxins.

Similarly, based on multiple lines of evidence, we propose that the identified DREPs encode unknown signaling peptides. First, all members of these families contain an N-terminal signal sequence, showing that they encode secreted proteins. Second, we identified enzymatic processing sites characteristic of signaling peptides (basic and dibasic amino acids). Third, evolutionary trace analyses show a pattern of conservation characteristic of signaling peptides (but contrasting to toxins). Forth, all five families are found throughout different classes of Mollusca and Annelida, and in one case also in other protostome phyla. Fifth, we observed expression of these genes in cone snail nerve ring tissue and, in most cases, also in neuroendocrine and/or secretory tissues of the mollusks *A. californica* and *D. pealeii*, and the annelid *L. rubellus*. Lastly, we show that one of the identified DREPs is a member of the established CHH family of signaling peptides. Jointly, these findings support that these families very likely encode novel signaling peptides. Future studies using MS/MS sequencing, e.g., from milking, will be needed to confirm the presence of the translated peptides, including potential modifications.

We have gathered several lines of evidence that the identified DREPs encode neuroendocrine signaling peptides of biological importance. However, in the absence of functional data, it cannot be ruled out that the peptides discovered here have alternative functions. Functional studies are required to establish the biological role of the identified DREPs ideally in combination with identification of their molecular targets. However, if, as we propose, these sequences encode signaling peptides, these peptides and the systems they regulate are likely of functional importance in prey. The evolutionary cost of producing toxins is high, and toxins that target systems of little importance should be selected against. We consequently propose that the signaling peptides identified here regulate critical functions in mollusks, annelids, and other organisms.

The new doppelganger/DREP pairs have already revealed several new insights into peptide evolution and putative function. Using structural homology searches we observed that one of the new DREP peptide families showed significant similarity to CHH peptides found in arthropods and nematodes – peptides that have been firmly established as signaling peptides (Chen et al., 2020). CHH was originally defined by its hyperglycemic activity (Abramowitz et al., 1944). However, it has become clear that CHH and its related peptides have a wide range of physiological functions in metabolism, water and ion balance, development, immune regulation, molting, and ovarian maturation (Chen et al., 2020). When we compared the gene structures (i.e., number, phases, and positions of introns) of the CHH DREP genes with those encoding arthropod CHH, we found identical patterns serving as evidence for the common ancestry of these signaling peptides. This finding on the existence of CHH outside of ecdysozoans expands our understanding of the evolution and functional importance of the CHH-family. While the structural similarity is high, the sequences show limited similarity and even have a different number of disulfide bonds suggesting that tertiary structure comparison could prove an important addition to signaling peptide research. Finally, it is notable that there are other examples of doppelganger toxins targeting this signaling system. Both venomous spiders, centipedes (Undheim et al., 2015), ticks and wasps (McCowan and Garb, 2013) have convergently evolved toxins that mimic CHH peptides further underlining that CHH and related peptides are functionally important.

Additionally, our discovery of exon shuffling in Hairpin doppelganger toxins is, to our knowledge, the first confirmed example of a conotoxin that has been recruited by this mechanism. Conotoxins are grouped into superfamilies that share extensive signal sequence identity, and the toxins within these superfamilies have a common genetic architecture ranging from 1 to 6 exons (Phuong and Mahardika, 2018). Here, we showed that cone snail Hairpin doppelganger toxins evolved by fusion of the first exons of contryphan toxin genes with the endogenous cone snail DREP and thereby adopting the contryphan signal sequence. It is likely that exon shuffling has been a driving force in the evolution of conotoxins. Recruitment of endogenous proteins to the venom system requires changes in the regulatory network of these proteins. Exon shuffling could constitute a process to rapidly change the tissue and level of gene expression of an otherwise lowly expressed endogenous peptide to acquire the necessary expression site and quantity to act as a toxin, as seen in other chimeric genes (Rogers et al., 2010; Rogers and Hartl, 2012).

In conclusion, this paper is a proof of concept for the use of doppelganger toxins to discover high likelihood signaling peptide candidates. We anticipate that toxins from other organisms can be employed in a similar way using the generalizable approach described in this paper. Venomous and poisonous animals are not the only example of organisms that have evolved molecules to disrupt the behavior and physiology of another. Both parasites and pathogens are likely to use doppelganger toxins to manipulate their hosts to their advantage. Recently, several hormone-like sequences were detected in pathogenic viruses (Altindis et al., 2018; Huang et al., 2019). We propose that, in the future, the method described here can also be used to identify such yet-unknown genes in parasites and pathogens and their hosts.

## Data availability statement

The datasets presented in this study can be found in online repositories. The names of the repository/repositories and accession number(s) can be found at: https://www.ncbi.nlm.nih.gov/genbank/, SRR22829302, SRR23242094-SRR23242120; https://www.ebi.ac.uk/pride/archive/, PXD038986, PXD038992, PXD038993.

## Author contributions

TK, BO, and HS-H contributed to conception and design of the study. RB and PS acquired data. KC assembled transcriptomes. TK, JT, RB, and PS identified toxins and DREPs. TK performed the analyses and wrote the first draft of the manuscript. All authors contributed to manuscript revision, read, and approved the submitted version.

## Funding

This work was supported by a Villum Young Investigator Grant (19063 to HS-H), a Starting Grant from the European Commission (ERC-Stg 949830 to HS-H), and a National Institute of Health Grant (GM048677 to BO).

## Conflict of interest

The authors declare that the research was conducted in the absence of any commercial or financial relationships that could be construed as a potential conflict of interest.

## Publisher’s note

All claims expressed in this article are solely those of the authors and do not necessarily represent those of their affiliated organizations, or those of the publisher, the editors and the reviewers. Any product that may be evaluated in this article, or claim that may be made by its manufacturer, is not guaranteed or endorsed by the publisher.

## Footnotes

^1^ roucoulab.com/en/downloads.html

^2^ https://github.com/Thomaslundkoch/toxmims

^3^ https://toolkit.tuebingen.mpg.de/tools/clans
