# Supplementary material for: A toxin-based approach to neuropeptide and peptide hormone discovery
Source: Front Mol Neurosci. 2023 Aug 31;16:1176662. doi: 10.3389/fnmol.2023.1176662 (PMC10501145; doi:10.3389/fnmol.2023.1176662)
Supplement: Supplementary file 10 [file Data_Sheet_10.DOCX]

Intron position and phases

===> Triangle <======

Toxin

>C.ventricosus toxin

MKAGDQLKMFFIFMCLWYVTDGQKCGRNPLIPIEAIAQKT - phase 1 6403bp

GKTTVYVLQVFRQDDPAQFEVWRQKFEAYNHCVGFLETGYYKRSLMEKSETSQDQLQRRGERGRELPGALQAAVGSSNTQRMLTGKRSVAAAAAGGGGDADGAAQGKSL

Conus

>C.ventricosus

MVKREDLLKIFFVYLCLLHVSPGHPCGRNPISRFQPSP -phase 1 6341bp

SRDINSLLNLLRSSNPALYQQVQDEWRRYADCVGLVDTGYFKRSSTGTSEAPQEHLQGRGQRAMALPEAFRAANRSRRTQRLLTLMRSLAAAAAAAGDGDGDAEGAAQALRL

Annelida

>ELT98797.1 hypothetical protein CAPTEDRAFT_191853 [Capitella teleta]

MNIAGLLFLLGALHWSTVAAQTYGEECGANPMLKAVLVARRA - phase 1 907bp

GRPLRQVLEVLRRIDPPAFKELAQKFRQYAACVALVDTGYFRKRSQASPVTRSNILVTTKTPSPSVNLGFLRKFQMKLRAWLQTKAVSKWPSIIN

>ELT98795.1 hypothetical protein CAPTEDRAFT_191851 [Capitella teleta]

MAHSLQMAVALVLTYSCCCNATAAVYGEECGSNPMQQVLMVARRS - phase 1 113bp

GHTLTHVLEVLRKIDPPAFRTLAQKFRMYAACVGLVDTGYFKKRSSMPSLLEEPARDFHRSQSRSVLSAEDEDYLPQEFASWLKKYSSSSASENSAYGSAREFGGSVDRFGDDLLELIHDEYPKRTQPITEQEHKELLSDFSDLRPFFVWK

Mollusca

>XP_034298906.1_uncharacterized_protein_LOC105337531_isoform_X1_[Crassostrea_gigas]

MVKKKDIVKVFVIYLSLCHVCPAHPCGENPLRRLISISFKT - phase1

GHPLRSVLLLLREMDGNVYKQLQVDFQRYAGCIGIVDTGYFKRSPAPDSFSKRTPQSSTGHNDGLELFKTWTNRN - phase2

RTSDRLSLLLEAQNSLSNELFPFSRRLQRRIR

>XP_025092781.1_uncharacterized_protein_LOC112563200_[Pomacea_canaliculata]

MVRKETLLKVFFIYLCFCHLVPGHPCGSNPLRRFRPLSRML - phase1

GKSIDTLLDLLRESNPALYEQVQLEWRLYAECVGLVDTGYFKRSSDDTQGRVDSEAGPLARSHDGDEGLAADDLLERVLQSWMDATSD - phase2

SDRDLDSGHL

Rotifera

>CAF1000162.1 unnamed protein product [Brachionus calyciflorus]

MKIQTVTIIILFCIAIDGTLSHTHPECGESPWLKYKVLATKI - phase1

GWEWGQLIRFMRTETTMEYNLLLKQVKEYADCIALSNIPL

>RNA38265.1 hypothetical protein BpHYR1_030566 [Brachionus plicatilis]

MVLNSKNKICVFFIMVMITVISKAKVNSLSFHPECGESPVYKYKELANAI - phase1

GWDINQLIRFMRAEKKEDYEKLIRAVKLYADCVALSQIPIK

Arthropoda

>XP_042220147.1 uncharacterized protein LOC121864977 [Homarus americanus]

MWTSTANTTPATVM - phase0

DRVWCVLWVGVVTVAGTGGRVMPLLQELEASDGCGPNPLTVVVAAGNDL - phase1

GHLEAVEVMAVLESDPETWGEFLALIHAYDDCVRTGQL - phase 2

RFKRMAPPPLRRSASLAGPAALRLHNASKSTSSDLSAQPRSSFSQQHLLYRLLQHQDPPSRSLDHHRHIQA

>EFX71890.1 hypothetical protein DAPPUDRAFT_308661 [Daphnia pulex]

MFAVLVSIHVVLLALAVAGLPAHHQLEARQSSDGCGPNPLVAVISYAREF - phase1

ADFSPHQLMRFIESDTRAGSQLRSLLRTYDDCVRTGDGTRYKKSGSALEMMDDVESDMGKSPFRMLAGNNNKSQQQQHSLQQANQSLLRALASHRLL

Tardigrada

>GAV04599.1 hypothetical protein RvY_14861 [Ramazzottius varieornatus]

MLNRLVFLTLLLAGVLVAKEMADGCGMNPLMALVAVADKT -phase 1

NQSVENLMGKFSADKMAFREFVMLLGRYESCIRVGSGTRYRKRSPEQQVADLIGLL

>GAV04599.1 hypothetical protein RvY_14861 [Ramazzottius varieornatus]

MLNRLVFLTLLLAGVLVAKEMADGCGMNPLMALVAVADKT - phase1

NQSVENLMGKFSADKMAFREFVMLLGRYESCIRVGSGTRYRKRSPEQQVADLIGLL

Platyhelmintes

>KAG5448219.1 hypothetical protein CSKR_105721 [Clonorchis sinensis]

MQFSFLIVTTSLISCIATASANVHCGRNPMSVFKRLVFEESNG - phase1

TIDPKFALGQLEIREPRTYTRLLKSHRDWLLCIQKTETGYFKREAFGSSQRTPTIQMRIDGKFEDDQ

>XP_009171979.1 hypothetical protein T265_08028 [Opisthorchis viverrini]

MMQFSFLIVTAGLLSCIVNTSANVHCGRNPMGVFKRLVFEESNG - phase1

TIDPKFALGQLEIREPRTYTRLLKSHRDWLLCIQKTETGYFKRAAFDSSQRTPTSIYGTFEND

====> CHH <=====

Conus

>ventricosus_1

MAVFGLALMTLMLLPESSYTLRHYAGK - phase 2

LPDGHDVTFYLHICSVCGEFYGGSVERNCIVDKSFETFYNCKAAILQRRRK

Molluska

>KOF87272.1 hypothetical protein OCBIM_22017144mg [Octopus bimaculoides]

MMMSLPRVAIIALICTLMCSYGSSMTLEEIRR - phase2

SIPKGATLYSYAGTCEACGEFYGSSYTYRCLTDKTFETYHKCNIAVNEKRK

>OWF53087.1 hypothetical protein KP79_PYT00509 [Mizuhopecten yessoensis]

MARVTCLLLLGCLLSCCMANTLESLVRR - phase2

NTPKGHTFSAFVEICESCGEYFGNSLSYRCIMDRSLTTFRQCNTAVVSVNRRR

====> Tail <=====

Toxin (no intron)

>C.ventricosus

MLSLGIIVLACLSMTRMITPMRAPPLVPSQVRTGPVRSTTGTTDTRGVTTITCTTPECVQIIQAYLKWREENGYGASNNRYG

Molluska (no introns)

>XP_025084112.1 uncharacterized protein LOC112558117 [Pomacea canaliculata]

MCPEVAMDRVTVLLLLLAMLHAASATSTANRTAASLLQLLLADGEGDSPAAPPFQPAPDATTSGCPLHHPDCSPDGDLPAHPREGQTHDLQAPLPASEDEVLIYGIPVKDIPPHLIPAALRKERLLYLSNLPDDVPRLRDGLDAAAFCFTQDCVEMMKEFDQWQREHGYGTPGGRWG

>XP_045210103.1 uncharacterized protein LOC123561658 [Mercenaria mercenaria]

MKLTLTMISALFCYQFTTALSLPLIEDNETQNSNDQDQQTHASQTSINTDTSLKSLIDSESEVYVDIGYSETTEKMNNSKSKLENKSNETEDDPLAGICQTPECIQQLQEYMKWRQDNGYPVPSGRWGK

Annelida

>ELT87057.1_hypothetical_protein_CAPTEDRAFT_197833_[Capitella_teleta]

MRPVLLFLLPLIIAAIFPPAETTNDVTEGYLDTKDEATVPTASIMPLIDGVPIDLIPDEFLRLLLLGINSELEDFDFHTNYCTTSACREILEEYEKWREENGYGKSEIR - phase2

YISVVG

====> Medial <======

Conus

>C.ventricosus

MGAPVLSRWLLLGFLLSLTLCQMPATARYTYRLQPYR - phase2

PAMTRRGCGGFPCAYNHLARLVGRFAMMRAIMRIMSDCAHDEHCSPG

Mollusca

>XP_005095677.1_uncharacterized_protein_LOC101858621_[Aplysia_californica]

MVRSTVLVAGSLVLVLSLVHLQVTDASDIDDVTRLLMQIDNVLA - phase2

KFRGKRGCIGYMCSYSHMSSSAGSKAVHNSLMKFLYNCAKDPHCSP - phase1

GKRKRRSVSETDTPLWSMLRNRDLETFLRRRDIKSLLSGSSSLNKAPS

>XP_033752434.1 uncharacterized protein LOC117336157 [Pecten maximus]

MDRVMSVKVTEILTISLFLLLTLRVTLAYPFGLQNFVVPKEPAADDQSEYW - phase2

NELLEDRGPSVPVKRGCVGFPCVYTHMAERAGRASIERYIAKLIEDCMNDDHCNP - phase1

GKRKRRSLLKQINQRR

Annelida

>Alitta virens genome assembly, contig: atg000332l_1, whole genome shotgun sequence

MNLLPGWFSLTIITLLGGLLHTYAYPTDNEESNYEIDTHLTVPDLSSLFEYEN - phase2

IPAEWPSPKTLPEKRGPCHGTACVYSHIGAKAGHSSLRRLKLTLLRECIDDPLCFAAGKTLR

====> Hairpin <======

Conus

>C.ventricosus

MVRAALSLLLLATLAVLVI - phase1 118,485bp

ANERAEAEEPQHHRAKRQDDMAAVDDYPLDDVDMMQRIFRTPLKRQWCRPGMSFNPVLGTCTLSLA - phase0 59,272 bp

ALRGRGRSFRGV

Mollusca

>XP_005089801.1_uncharacterized_protein_LOC101860216_[Aplysia_californica]

MGSGSRLVCLLLLSMLALAAVS - phase1

ASSLDQTRSRRSLQAADSSDDMLADDWAASVPTDDLLMRLGEGREKRGWCRPNMTFNSILGRCTYVYS - phase0

KLKGGRGKRGWCRPDMIFNPILGRCTFTYA - phase0

AIRNRGRG

>KAG5691889.1 hypothetical protein BaRGS_033252 [Batillaria attramentaria]

MKL - phase1

GQTEAAAGRESRRVKRQGYNSFDSDLGEYPQYDVDLMQRVFKTPLKRQWCRSGMSFNPVIGTCTLSLA - phase0

FLLS

Annelida

>ELU17817.1 hypothetical protein CAPTEDRAFT_221947 [Capitella teleta]

MCNADAMRQTT - phase1

LLGKGMLLDDEEFPPAPQMESKDMMAALLAGWSSNQDDEYQRMNPRTEGDGIQRSKRYFCRKFFVYNPVRGRCQPTPQ - phase0

RRLLRYSDWVHLRRLWQGGGVMQDWNSEWAAN - phase1

SRKVSKITGRKQVVNVEGSLSAASELIIMRSASGVDLGPLLFNSPPYFTHLMCGSVRKRMNSNCNATV

>GINI01011046.1 TSA: Alitta virens c14478_g1_i1, transcribed RNA sequence

MKNVIVTLFAALLVCLLLCGR - phase1

TACGVIKRDTESVPYEFIEENQALEEEEPQFGDLDLPTKRHLCRRYFIYNPVKGRCMPTLM - phase0

AFRAMRSKSLRSFPNPQAWAHFG
