# Supplementary material for: A toxin-based approach to neuropeptide and peptide hormone discovery
Source: Front Mol Neurosci. 2023 Aug 31;16:1176662. doi: 10.3389/fnmol.2023.1176662 (PMC10501145; doi:10.3389/fnmol.2023.1176662)
Supplement: Supplementary file 12 [file Data_Sheet_12.DOCX]

Supplementary file 10. Alignment of endogenous cone snail DREP transcript with doppelganger toxin transcripts. Translations into peptide and toxin precursors are placed above or below the alignment, showing that there is significant sequence similarity of both the 3’ and 5’ UTRs. (A) DREP-1, (B) DREP-2, (C) DREP-4. An alignment of DREP-5 with endogenous gene is seen in Supplementary file 11. As we did not identify any transcripts of the endogenous gene for cone snail DREP-3 this family is omitted.

(A) Triangle DREP

textileNR.TRINI TTGAAGAAGTAAAGTTTTGCAGGGTGAAAAAAAAAAAAGAAAAAAAGAAAATCGATC---

rattus.TRINITY_ -------------------------------GAGGAAAGCTTAAAATACGAGTGATCGGC

.*..**** **** * .* .****

textileNR.TRINI ---------------------------------------TGACAGCTTTTGAAGTATCGG

rattus.TRINITY_ TCTACCCCCTCCAAACCTCAACATTTCTCCTCACTTGGTTGCAAGCTTTGGGACCATTCA

** ****** *.* .**. .

textileNR.TRINI TCGTAAGAA------------CGAAGATGGCAGCGATGAAGACGACGATGAAGACGACGA

rattus.TRINITY_ TCACCAGGGCTTGTTCTCAGCCGGAAACGTGAGGAATACAGGTAAAGG-AGATCCAGTGG

**.. **.. **.*.*.* ** .**. **...* *. ..* *...*.

textileNR.TRINI GGAAGACGTTTGACG-----AAGAGGGGGACCAGAAGGCGAGGGAAGAGGAGGAGCACTG

rattus.TRINITY_ CGTGAACGATCGACGACACCAAGCAAAGAAAAAGATACAGAGCGACTGGGTGAGACAGTG

* ..*** *.**** *** ...*.* *** . *** ** .** *...** **

textileNR.TRINI CGTCAAGAAA-----TGGCTGGAGTC--------------ACTCGTCAGTGGTGCGC---

rattus.TRINITY_ CAATAACAGATCTGTTGTTGGGAGTCTGAGGAACTGCATAGCTCATCAGAAGTGCGCGTG

*. .** *.* ** . ****** .***.**** .******

M V K R E D L L K I F F V Y L

textileNR.TRINI -TGTCGTTCAGGAGGATGGTGAAGAGAGAGGACCTGCTGAAGATTTTCTTTGTCTACCTG

rattus.TRINITY_ TTGTCATTCCGGAGGAGGATGAAGACTGGAAACCAGTTGAAGATTTTCTTCATCTACCTG

****.*** ****** *.****** *...*** *.*************..********

M K T G N Q L K I F F I Y L

C L L H V S P G H P C G R N P I S R F Q

textileNR.TRINI TGTCTGCTGCACGTCAGTCCCGGCCACCCGTGCGGCAGGAACCCCATCAGCCGCTTCCAG

rattus.TRINITY_ TGCCTGTGGTATGTCGCCACTGATCACATATGTGGTGAGAATCCTCTCATCC--------

**.***. *.*.***. . *.*..*** ..**.**...***.**. *** **

C L W Y V A T D H I C G E N P L I

P L T K M L G R D I N S L L N L L R S

textileNR.TRINI CCGCTCACCAAGATGCTAGGCCGAGACATCAACTCCCTGCTCAACCTCCTGCGCTCCT--

rattus.TRINITY_ CAATTATACAGATTGGCAAACAAAGAGGTGTATCCTTAGTTCGGGTGCTCACGCTCTTGA

* ..* **.. ** .*..* .*** .* *..*.. *.**.. . *...*****.*

P I I Q I G K Q R G V S L V R V L T L L

S N P A L Y Q Q V Q D E W R R Y A D

textileNR.TRINI ------CCAATCCCGCCCTCTACCAGCAAGTGCAAGACGAGTGGCGACGTTACGCCGACT

rattus.TRINITY_ AGAACACGGATCCCACTGCCTATAACAAGTTGCGTGAAGAATTTACTGCTTACATCCAGT

* .*****.*. .***. * *. ***. ** **.* ****..* * *

K N T D P T A Y N K L R E E F T A Y I Q

C V G L V D T G Y F K R S S T G T S E A

textileNR.TRINI GCGTGGGCCTAGTGGACACCGGCTACTTCAAGAGGTCCTCGACGGGCACTTCAGAGGCCC

rattus.TRINITY_ GCGTGGGTTTAAGAGACACTGGCTACTTCAAGAGGTCCTTGACGGAAAAATCCGCGACTT

*******..**. .*****.*******************.*****. * ** * *.*..

C V G L R D T G Y F K R S L T E K S A T

P Q E H L Q G R G Q R G M A L P E A F R

textileNR.TRINI CCCAGGAACACCTTCAAGGACGTGGACAGAGGGGGATGGCACTGCCAGAGGCCTTCCGAG

rattus.TRINITY_ TCCAGGACCAACACCAGTGGCGTGGACAGCGGGGTCTGGAGCTACCAGGGGCGTTACAGG

.****** ** * .**. *.********* **** *** .**.****.*** ** *..*

F Q D Q H Q W R G Q R G L E L P G A L Q

A A N R S R R T Q R L L T L M R S L A A

textileNR.TRINI CAGCCAACAGAAGTCGCAGGACCCAGAGGCTGCTGACCCTGATGCGTTCCCTGGCTGCTG

rattus.TRINITY_ CAGCAGTCGGAAGCTCCAACACCCTGAGGATGCTAACTGGGAAGCGTTCTGTGGC-----

**** . *.****.. **. **** **** ****.**. ** ******. ****

A A V G S S N T L R M L T G K R S V A

A A A S A G D V D G D A E G A A Q A L R

textileNR.TRINI CTGCTGCCAGTGCTGGTGATGTTGACGGTGATGCTGAGGGTGCTGCCCAAGCATTGAGAC

rattus.TRINITY_ ----------TACTGGTGATGGTGGAGGAGATGCAGACGGTGCTGCCCAAGGAAAGAGCC

*.********* **. ** ***** ** ************* * *** *

T G D G G G D A D G A A Q G K S

L

textileNR.TRINI TCTGAGGTTCCTGGGTTCCGGTTCTTGTTCCTGTCCCGGTGCCTGAGGGAGACATGCTCC

rattus.TRINITY_ TGTGAGGACTCTGGGT-----TTCCTTCTCCTCTACCACGGTTAATGGAGAGTCCGGCGA

* ***** ..****** ***.* .**** * **. *.. . **..... .* .

L

textileNR.TRINI TCATAGTTTCATGGATGAGGAGGCCATTGAACAATGACCCCCAGCACCCAGTCTGCTACG

rattus.TRINITY_ CCATGAT-------------------TTGAACA-----TCACTGTACCCAATCTGCTCAG

.***..* ******* .* * *.*****.****** *

textileNR.TRINI CCAAACCACGTCATCTACACACGCTACACTGGAATCATCGCTGCGTCTGGAGTATATGAG

rattus.TRINITY_ CTCATTTTCTT--TCTAAACGTCTTCAACTTGAGTCGACGCGACGAGAAAAAAACAGAGA

*. * .. * * **** **.. .* *** **.**. *** .** ..*. *.* ...

textileNR.TRINI TAACGCCCAGAAT-------ATGTACCAATCACGGGACATGACTATCTACAGTGAACGGA

rattus.TRINITY_ CTGTCTCAAAATTTCCAATGGAGGAATAATCCTCAAAAATGTAAGCCTTCATTCACGTGA

. .. .* *.* * . * * .**** . ..* *** ..** ** * * **

textileNR.TRINI AGACAAAAAAAAAACCCACAGCCACAATCTAATGGCGGTATTTAGCTGGAAAGGAAAGAA

rattus.TRINITY_ AGATGAAAACGTCAGCGTCTTTCTTTACCACCTGGTAGACTTCTACCTG-----------

***..**** . * * * .* . *.* ***..* **. .*. *

textileNR.TRINI GTCCAACTTCTGGCCATGAAATGTAGCATTAATCTTGTTTAATAT----------ACCGA

rattus.TRINITY_ --CTGTACATTAGGCATGTTGTGAGCTAATGGTTTCTTTGAACATTACGCATAAAACTGA

*.. . .*.* **** .** . .* *..*.*. ** **.** **.**

textileNR.TRINI AGAATTGGATT------CTCTACGTACAGAAAAAAAAGTTGTTAAATATTCTCTCTCTCT

rattus.TRINITY_ AGAAATGATATGAATAACTTTATTTTGGAAGAAAGAAATGTAACAACATTTTACATATCG

**** **. * **.**. * ..*.***.**.* **.***.* . * **

textileNR.TRINI CTCTCTCTCTCTCTC---------------------------TCTCTCTCTCTCTCTCTC

rattus.TRINITY_ TTAGCACATGAATTCGTGACAGATGTGACTCGGCGGAAATAAATTCAAACTACAACTCTG

.* * * . .** .** ** . ****

textileNR.TRINI TCTCTCT

rattus.TRINITY_ AAAAAAA

====================

(B) CHH DREP

textileNR.TRINI AACTCTTCTTCCTTCTCCTTCCTTCTTTCATTCCTTCCTCGTTCCTTCCTCCCTACAGTG

textile.TRINITY GATCGTGC----------------------------------------------------

.*.. * *

textileNR.TRINI CCAGAGAGACTCCACACCTACCTACTACTCCCATATCCACACCGCCACCACCACCACCAC

textile.TRINITY ------------------------------------------------------------

textileNR.TRINI CACCACCACCCCATTCTACCCTGTTGGTCTGGCTTACAGCTCAGGAGGCAGAAAGGAAGA

textile.TRINITY ------------------------------------------------------------

textileNR.TRINI GAGAAAAAGTCGGGAAGGAGGGAGCCTGGTGAGAGAGACAAAGCAAAGCAGAGCCAAGCA

textile.TRINITY ---------------------------------GGAGACGAAAC----------------

.*****.**.*

textileNR.TRINI AGCAACTAATCCAGCTGACCTGAGGGTCTTGATTTCATTCACCGACGCTTTCATCCTTAT

textile.TRINITY ------------------------------------------------------------

textileNR.TRINI TTCGGTTCGTTCCGGCGCCCAGGTCGTTCCCGTCCGTCGTTTTGAGACTCCCCCTTTTCC

textile.TRINITY ------------------------TGACCTCGGTTATCTTGTTAATTCCACAACTTGATC

.* .*.** ...** * **.* *. * *** .*

M K T M A V F G

textileNR.TRINI GGCGGAAGTTCCGGTTCTCTTCGGTCTCCCCCAGAAGATGAAGACTATGGCTGTCTTTGG

textile.TRINITY GAGTCGAGT--------------GTTCCCCCGTCATAACGTGTAGTATGGTTGTCTTTGG

*. .*** **..**** * .*.* . * *****.*********

M V V F G

L A L M T L L L L P E S S Y T L

textileNR.TRINI CTTGGCACTGATGACT------------CTGCTGCTGCTGCCAGAATCCTCCTACACCCT

textile.TRINITY CTTGATACTGATGACTCTACTGCGGCTGCTGCCGCTGCTGGCAGAATCCCCCGACTACCC

****..********** ****.******* ********.** ** **.

L I L M T L L R L L P L L A E S P D Y P

R H Y A G K L P D G H D V T F Y L H I C

textileNR.TRINI ACGGCACTACGCTGGAAAGCTTCCGGACGGCCATGACGTCACGTTCTACCTTCACATCTG

textile.TRINITY AGAGCATTATGCTGGAGAGATTCCGGACGGCTATGAAGTCGAGTTCTTCCTTCACATCTG

* .***.**.******.** ***********.**** ***. ***** ************

E H Y A G E I P D G Y E V E F F L H I C

S V C G E F Y G G S V E R N C I V D K S

textileNR.TRINI CTCCGTGTGCGGCGAGTTCTACGGTGGCTCAGTGGAAAGGAACTGCATCGTGGACAAAAG

textile.TRINITY CTCCGTGTGCGGCGAGTTCTACGGTGGCTCAGTGGAACATGACTGCATATACGACAAAAC

************************************* . .******* *******

S V C G E F Y G G S V E H D C I Y D K T

F E T F Y N C K A A I L Q R R R K

textileNR.TRINI CTTCGAGACCTTCTACAACTGCAAGGCCGCCATCTTGCAACGACGCCGGAAGTAGTTCCG

textile.TRINITY CTTCGAGACCTTCTTCAACTGCAAGACCGCCGTCTATGATCGACGCCGGAAGTAGTTCCG

************** **********.*****.*** * ********************

F E T F F N C K T A V Y D R R R K

textileNR.TRINI GGACAGGAAGTGTCTACCTGGCCCGAGGATCGGAAGTACCTGGCACAATGGGCGTGTAAC

textile.TRINITY GGACAGGAAGTGTCTACCTGGCCCGAGGATCGGAAGTACCTGGCACAATGGGCATGTTAC

*****************************************************.*** **

textileNR.TRINI AAGATTCCTGGTGATAGATTTCAACTGTTACATAACTATTAATACCATTTAGATTTTGTT

textile.TRINITY AAGATTCCTGGTGATAGATTTCAACTGTTACATAACTATTAATACCATTTAGATTTTGTT

************************************************************

textileNR.TRINI AAAAAAG----CAAAACCTGAAATGAATTCAGTCTGAAATTTGTGTGTGTGATTTGTGTT

textile.TRINITY AAAAAAAAAATCAAAGCCTGAAATGAATTCAGTCTGAAATCCTTGT--------------

******. ****.************************.. ***

textileNR.TRINI TTGTTTTAAAGCTGGAAGAAAAAAAAAACACGTAGTGTGTCCTGAAACAAAAGTTTGTTG

textile.TRINITY ----------------------------------GTGTGTCCTGAAACAAAAGTTTGTTG

**************************

textileNR.TRINI ACGAATTAGGCATTTTCAGTCTTAATAACGAAATCAGAAGAAAGGGGAAAAAAACGACAG

textile.TRINITY ACGAATTAGGTATTTTCAGTCTTAACAACGAAAACAGAAGAA--GGGGAAAAAACGACAG

**********.**************.******* ******** ***.************

textileNR.TRINI AAAAGAACTAATGTTCATGGCCCAAATTGTTGTAAAATGTGAATCGAACAGTTGTTTGTT

textile.TRINITY AAAAGAGCTAATGTTCATGGCCCAAACTGTTGTAAAATGTTAATCGAACAGTTGTTTGTT

******.*******************.************* *******************

textileNR.TRINI TGTTTCCAAGCGTTCTGTCATGAATGTTAATTTACAAAGAGGCATTAGGACAATGGAACT

textile.TRINITY TGTTTCCAAGCGTTCTGCCATGAATGTTAATTTACAAAGAGGCATTAGGACAATGGAATT

*****************.****************************************.*

textileNR.TRINI GTAAACAAATTAC----AAATGAATAAAACTTCGTGCG-------------

textile.TRINITY GTAAACAAGTTACAAATAAATAAATAAAACTTCGTGCGCAAACAAATCGAG

********.**** ****.****************

=======================

(C) Medial DREP

textileNR.TRINI AAGAGATCGAGCACGAAAGGAAGAAAAATAGAGAACGCTACAAGGAAGGAAAAAAAAAGG

14357X3.C.furvu ------------------------------------------------------------

textileNR.TRINI AAACAACAAAGACAAAGTGCATAACTGAAGGAAAGAAGGAAGTAACAAAATAATAATAGT

14357X3.C.furvu ------------------------------------------------------------

textileNR.TRINI GATAATAACTAACTAAATAGAAAAAGAAGCCACCTAGAAAACATCGGCTGAAGAGGACCA

14357X3.C.furvu ------------------------------------------------------------

textileNR.TRINI AGAAATAACAAGAGAAATATCACAACTTTTCTCAAACTGAAGCTGATCTCATCTATTTTC

14357X3.C.furvu ------------------------------------------------------------

textileNR.TRINI TTCTGTATCAACGAAGAAGGGGTAAACCCAAACATAGGAAACATTACGTCGCTGTCTTTT

14357X3.C.furvu ------------------------------------------------------------

textileNR.TRINI CCCATATTTTTGAACTGACAAGACAAAAAAAGCCAGCACTGGGGAAAACATAAATATTAA

14357X3.C.furvu ------------------------------------------------------------

textileNR.TRINI AACAGCATCAACGCCACTATACCACTCTGCTGATCCAGCCTTTTTTCTATCTATCCTCTG

14357X3.C.furvu ------------------------------------------------------------

textileNR.TRINI CAGTTTCCCCCCTCAGTCGTCATCTTCCCAGCCTTCAGCAAAAGACGGAAGCAGGTGGCT

14357X3.C.furvu ------------------------------------------------------------

textileNR.TRINI GATGCAACAGGCTTTCCCCACGCTAACTAGCTCAAGGCCTAAAAGCTTCGCACCGCTTCG

14357X3.C.furvu ------------------------------------------------------------

textileNR.TRINI CCTGTACAGAACCACTCCAAACAGCTCTGTAGACACACCCACTTCTCTTCAAAAACAGAG

14357X3.C.furvu ------------------------------------------------------------

textileNR.TRINI GGAAAATAAAAAGCCAGGCGTTTCTAAACTCGATTCAACGGTGCAAGACACGCCAGTGAG

14357X3.C.furvu ------------------------------------------------------------

textileNR.TRINI GACGTGAGGAGGTGTAGCTTTCTACCTGCAGCGCGGCGGACTCCCCCCCACCTCTAGCAG

14357X3.C.furvu ------------------------------------------CACCCCCACCCCTAGCAG

* ********.*******

textileNR.TRINI TGAAGACTACACGTAAGCTACTCCCCTGTCTGCGAGCGACACGCGCCCTCCGGATTTATC

14357X3.C.furvu TGAAGACTACATGTAAGCTACTCCCCTGCCTGTGAGCGACACACACCCTCCGGATTTATC

***********.****************.***.*********.*.***************

textileNR.TRINI AGCAGATCAAGTGAACAGCGAGATTTCCCTGATATCAGCGAGCTCCTGTCGCCCCCAACA

14357X3.C.furvu AGCAGATCAAGTGAACAGCGAGATTTCCCTGATATAAGCGAGCTCCTGTCGCCCCCAACA

*********************************** ************************

textileNR.TRINI GACACACTCACCGCCGCACGTCTCAAGCGCACAGCGACGACCTTCTCTCGTAGCCGGCCC

14357X3.C.furvu ATCACATTCATCGCCGCACGTCTCCAGCGCACAGCGACGACCTTCTGTCGTAGCTAGCTC

. ****.***.************* ********************* *******..**.*

textileNR.TRINI CTGAGTCGGAAATCACCGGTAGTTTTATCACTCGAAGGGAACAACCAAGACGGACCCACG

14357X3.C.furvu CTGTGTCGTAAATCACTGGTTGTTTTATCACTCGAAGGGAGCAATCAAGAC-GACCCACA

*** **** *******.*** *******************.***.****** *******.

textileNR.TRINI AACTCCACACACACACACACACGTCTTCACAAAAACCAAAGTTCCAGAATCTGATACCGT

14357X3.C.furvu AACTC--CACACACGCACACACGTCTTCACAAATACCAAAGTTCCAGAATCTGATACCGT

***** *******.****************** **************************

textileNR.TRINI TTTTCGTTCCCGGAGTTCTTCACAGAGACGATTCGTTTGTTTCTGAAGATTCCAGCTGAG

14357X3.C.furvu TTTTTGTTCCTGGAGTTCTTCACAAAGACAATTCGTTTGTTTCTGAAGATTCCAGCTGAG

****.*****.*************.****.******************************

textileNR.TRINI AAAACCGACAAAACGTGATTTCGACACCACCTTTTGTCTTCCAAGAGTCAGTTCAAAGGA

14357X3.C.furvu AAAACTGACAAAACGTGATTTCGACACTACATTTTGTCTTCCAAGAGTCAGTTCAAAGGA

*****.*********************.** *****************************

textileNR.TRINI AGCTGAGAACGTAGCGCGAGTGAACCTGAACGCTTGAGTGTTGTCGAGTTTGATCAACAA

14357X3.C.furvu AGCTGAGAACGTAGCACGAGTGAACCGGAACACTTGAGTGTTGTCGAGTTTGATCAACAA

***************.********** ****.****************************

M A M M R L C S W L L V A M L M S L

textileNR.TRINI TTCAAGGATGGCAATGATGAGGTTGTGCAGCTGGTTGTTGGTGGCGATGCTGATGTCCCT

14357X3.C.furvu TTCAAGGATGGCAACGATGAGGTTCTGCAGCTGGTTGTTGGTGGCGATGCTGATGTCCCT

**************.********* ***********************************

M A T M R F C S W L L V A M L M S L

T V C E A R G L P R R S A Q S G D M I A

textileNR.TRINI GACCGTGTGTGAGGCGCGAGGTCTTCCTCGGAGATCAGCGCAGAGTGGCGACATGATCGC

14357X3.C.furvu GACCATGTGTGAGGCGCGAGGTCTTCCTAGGAGATCAGCGCCGGGCGGCGACATGATCAC

****.*********************** ************ *.*.************.*

T M C E A R G L P R R S A P G G D M I T

F N V E A W L Q L M R E K I K Q L Q M A

textileNR.TRINI CTTCAACGTGGAAGCCTGGCTGCAACTGATGCGAGAAAAAATCAAGCAGCTGCAGATGGC

14357X3.C.furvu CAACGACGCAGAAGCCTCGCTGCAACAAAGTCGAGAAGGAATCAAACAGCTGAAGATGGC

* *.***..******* ******** .* ******..******.****** *******

N D A E A S L Q Q S R E G I K Q L K M A

V E S S K R G C N G F P C M Y T H L G A

textileNR.TRINI GGTGGAGTCGTCCAAACGAGGCTGCAACGGTTTCCCCTGCATGTACACCCATCTGGGCGC

14357X3.C.furvu GGTGGAGTCGTCGAAACGAGGCTGCGAAGGTTTCCCCTGCATGTACACCCATCTGGGCGC

************ ************.* ********************************

V E S S K R G C E G F P C M Y T H L G A

K A G R Q A L M K T L L S K L S D C A H

textileNR.TRINI CAAGGCCGGCAGGCAGGCCCTCATGAAGACCCTGCTCTCCAAGCTCAGCGACTGTGCCCA

14357X3.C.furvu CAAGGCCGGCAGGCAGGCCCTCATGAGGGCCCTGCTCGCCAGGCTCAGGGAGTGTGCCCA

**************************.*.******** ***.****** ** ********

K A G R Q A L M R A L L A R L R E C A H

D P K C S P G K R R R R S A P A Q A V V

textileNR.TRINI CGACCCCAAATGCTCCCCAGGCAAACGGAGACGACGAAGCGCCCCTGCCCAAGCCGTCGT

14357X3.C.furvu CGACCCCGCTTGCTCCCCAGGCAAACGGAGACGACGAAGCGCCCCTGCCCAAGCCGTCTT

*******. ************************************************ *

D P A C S P G K R R R R S A P A Q A V L

S L L T R D E R T T S

textileNR.TRINI GTCCCTGCTCACGCGAGACGAGAGAACAACGTCGTGATTATGACGTCATCTTAGTTCTAG

14357X3.C.furvu GTCCCTGCTCACGCGAGACAAGAAAACAACGTCGTGATTATGACGTCATCTTAGTTCTAG

*******************.***.************************************

S L L T R D K K T T S

textileNR.TRINI GCGGGTCTTTTCCAGATTGTTTTTCCAGTTCTGTGATTGGGTTTGTAAATGCAGCCCTTC

14357X3.C.furvu GCGGGTCTTTTCCAGATTTTTTTTCCAGTTCTGTGATTGGGTTT-TTAATGCAGCCCTTC

****************** ************************* * *************

textileNR.TRINI AGCGAACTGCCACGATGAGGACGCCATCCACGTGACTCGCCAGGATACCCTTGTGATAAC

14357X3.C.furvu AGCGAACTGCCACGATGGGGACGCCATCCACGTGACTCGCCAGGACACCCTTGTGATAAA

*****************.***************************.*************

textileNR.TRINI AGAAAGACTTAGAGAACAATGATAGTGATAATCACATGACAGGCATTATTCATTAGTCCA

14357X3.C.furvu AGAAAAATGTAGAGAACAATGAAAGTGATAATCA-ATGACAGACA-TATTCATTAGTCCA

*****.*. ************* *********** *******.** **************

textileNR.TRINI AAGATATCGGTGACTATGAGGATATTTTCTTCTTAGAAGTGTTACTTTCTTTGCCCCTTT

14357X3.C.furvu AAGATATCGGTGATTATGAGGGTATTTTCTTCTTAGAATTGTTACTTTCTT---------

*************.*******.**************** ************

textileNR.TRINI TTGTGGAAGGAAATGTTGGCGCGGTTTACATCACGTGAGGTTGTTTTATTCAGACGTATC

14357X3.C.furvu ------------------------------------------------------------

textileNR.TRINI AGTGACAAAACAGATGTATCCAAAACAGGTTCTGCCTATGTCTAAGTTGTACGCTTACTT

14357X3.C.furvu ------------------------------------------------------------

textileNR.TRINI TGCACCGCAGTCTCATGTGAATCTTCACTGTTTTGTTCGTATCGGTAATCTGATCGTCCT

14357X3.C.furvu ------------------------------------------------------------

textileNR.TRINI TGACCACTCAGTAGCGACACTTACACTTGCGTTGTTTCCCTCCCGTATTTTGTTTTCTTT

14357X3.C.furvu ------------------------------------------------------------

textileNR.TRINI ATTTATTAAAAGATTGGACAAAACAATCAGATTTGATAAGCTCCTCTTGGTGTGATTTCA

14357X3.C.furvu ------------------------------------------------------------

textileNR.TRINI CTTCACCACCCCTTACCTCAAACCCTTCTCCCCTGTTCACACACACACACACACACACAC

14357X3.C.furvu ------------------------------------------------------------

textileNR.TRINI ACACAATTCTTATCCTTAAGACAAAACGCAGAAGCAAACCACAAGTCAATGTTTCAAAAA

14357X3.C.furvu ------------------------------------------------------------

textileNR.TRINI GGTATTGTGTTTTCGTCCTGTTGCACTATATATATTTTTTGTGTGTGTGTTGTTAAAAAC

14357X3.C.furvu ------------------------------------------------------------

textileNR.TRINI AAATGCAGAGTCCGCACCCGGTAAAAAATACTCCTACACCGCCAGATATCATCATCGAAC

14357X3.C.furvu ------------------------------------------------------------

textileNR.TRINI ACGATGGGAAAAACCACGAGAAAAAAATACCAAAGCATGCTGAAAGAATTACGACTCTAG

14357X3.C.furvu ------------------------------------------------------------

textileNR.TRINI TTCAAACCGGGAAAGTACATCTCATCCTCAATCCAGATTTTCTTTTTTTTGTCATGGTTT

14357X3.C.furvu ------------------------------------------------------------

textileNR.TRINI CAAGTACAGCTGTGAGACTTCCTAAATGGTTACCGTTCGCTGCACCGAACCTCCCCCTTC

14357X3.C.furvu ------------------------------------------------------------

textileNR.TRINI CCCACCCATACCCTACCCTACTCTCTCTTTCCGTCCCCCTACCCCTGTCTCC

14357X3.C.furvu ----------------------------------------------------
