# Supplementary material for: A toxin-based approach to neuropeptide and peptide hormone discovery
Source: Front Mol Neurosci. 2023 Aug 31;16:1176662. doi: 10.3389/fnmol.2023.1176662 (PMC10501145; doi:10.3389/fnmol.2023.1176662)
Supplement: Supplementary file 13 [file Data_Sheet_13.DOCX]

**A**

C.rolani.NR.TRI MVRAALSLLLLATLAVLVI--------AHERAEAEEPQHHRAKRQDDMAAVDDYPLDDVDMMQRIFRTPLKRQWCRSGMSFNPVLGTCTLSLAALRGRG------RSFRGV

14357X3.C.furvu M--GKLTILFLVAAALLSIQVMVQGDGAHERAKAEEPQHHHAKRQD---GTDGYPVDDVDMMQRIFRNPLKRQWCRVGYAYNPVLGRCTISLSRIKYPGLYEIYEETRRSQ

* . *::*:*.: *:* * *****:*******:***** ..*.**:***********.******** * ::***** **:**: :: * .: *.

**B**

>C.rolani.NR.TRINITY_DN11383_c0_g1_i1 Entry:1888.conotoxin.O2 len:1839 tpm:372.48

GTCAGACTGCCAGGATATATAAACCTGAGCCCCCATTCCTGGCCAACCGACACGCCTCCAGCTCCTCTCCCTCTCACTCTCCACCTCTGACTCCATCGCTGTCCTTCCAGAAGCTCTCTTGGCATCAGCCAGGGAAACACTCTTGGGTCAATATCTGGCTCTCTGTCAACGCCCGGCTGCGGCCCTCCGCGAGAAGCTAAGCTAACAAACAGCCTTGTTGCAATCATCGGTGCGTGGAGAAGACGGTAGACACTTTTCATTGACGTTCTTGACAGCCTTCTTCAAGGGGTCATCCGTACAACAGAGGTCTTGAGGGTCGAGGCTCTTTGCTTGGAGAACAAGTAGTAACAGGGCGTGCAGATCAGAGGTAAACGTTTGGGAGGCAGTTGTCGTGGGACGCACCCCTCCCGGAAGAAGTGGACGACCTGACGAGACAGTCTGTGAGTCATCTTCCGAAGGTCAGACACAGGCTCATCATGGTCCGAGCTGCTCTCTCCTTGCTGCTGCTGGCTACACTCGCCGTTCTCGTCATCGCGCATGAGCGAGCGGAAGCGGAAGAGCCACAGCATCACCGCGCGAAGCGACAGGACGACATGGCGGCGGTGGACGACTACCCTCTGGATGACGTGGACATGATGCAGCGTATTTTCCGCACCCCTCTCAAACGTCAGTGGTGTCGGTCTGGCATGTCCTTCAACCCCGTGCTGGGCACCTGCACGCTCTCTCTGGCGGCGTTGCGAGGGCGAGGCAGAAGTTTCCGTGGCGTCTGAGAGCCAGCAGAGGGCGCCAGTGATGACCCATACATGCGTGGTCCTGCCGACCGCTCTTGCCCAGTCTGCTGTGCTTGTTTTGTCATTCGGTCACTAGAACCTGTCACGTTTTGGTTTACTTCCCTCCACCCCTCCTCCCCTTCATCCTAGTCCCCTTCCACGCCCACCTCACCTACCACCTCTCCCACAAACCCCATTGATTTCCTCCTAGTGTGTGTCCAGTTGTCATCAATGTGTTAAAGCCAGGTCAACCCAGAGTTGTAGTCACGAAGGTACATTCATCCATGTGTTAAAGCAACGTGAATCTTTAGTTCAGTTCACGAAGGTACAGTCACTAATGGGTTAACGCGACTCGAATCCTGAATCTGTTTACAAAGATACGGCTATCAATGTGTTAAAGAAAAGTAAACCCTGATACAGTCACCTGAGTGTTTTAGCAATATGAATCCTTAGTTCTGCTCACGAAGGTACAGCAGTCAATGCGTTAAAGCAATATGAATTCTTACTCCTTTTCACGATGTTACTGTCATCCATGTGTTACAGCAAGATAACCCTTAGTCCTGTTCACGATGTTACTGTCATCAATGTGTTACAGCAAGATAAGCCTTTGATCTGTTCACGATGTTACAGTCATCAATGTGTTAAAACAATGTAAAACTTAGTCACGTTCAAAATGATAGGAGTCTTTAACATGTCAAAGTAGAACAAGTTCTGACCTATGTTCAGGATAACTGGGTCATTGAAGTGTCAAAACAAACAACACCCTAGGCTTTCTCCAAAATAACCTGCCCACTAAGGTGTCAAAGCAAAATAAACTACTAGCACTATTTACGTCGCCCAGTAAGATGTTGAAATAACTCTTAGTTCTCTCCAACCTCATGAATTACATTCACGATAGTGCAGTCGTCAAAGAGTTAAAGCTCAAATAAACTTTCGGCTTTCTTGCAGATAGTATTTGGGCTTGATATGACTTCACCAGTAGACACAATGTTGCCACTTCCCCCACCCCCACCCCTACACTATTGCTGATAGGAATTTCACAAAGGAATAAAACTGAAATGAGATACAT

>14357X3.C.furvus.TRINITY_DN4643_c0_g1_i1 Entry:1888.conotoxin.O2 len:598 tpm:10665.97

CACTCTCTGTCTCCCTGATTGCTGCCTTCAGTCGACCCGCCCTCGTCGCCGCGCAGACCTGGTAAGAAGTGAAGAACCTTTATCATGGGGAAACTGACAATACTGTTTCTTGTTGCTGCTGCACTGTTGTCGATCCAGGTCATGGTTCAAGGTGACGGAGCGCATGAGCGAGCGAAAGCGGAAGAGCCACAGCATCACCACGCGAAGCGACAGGACGGCACGGACGGCTACCCTGTGGATGACGTGGACATGATGCAGCGTATTTTCCGCAACCCTCTCAAACGTCAGTGGTGTCGGGTTGGCTATGCCTACAACCCCGTGCTGGGCAGGTGCACGATATCTCTGTCGAGGATCAAGTATCCTGGGTTATATGAAATATATGAAGAAACCAGAAGGAGCCAGTGATGACCCATACATGCTTGGTCCTGCCGACCACTCTTAACTTTCGGCTTTCTTACAGATAGTATTTCGGCTTGATATGACTTTACCATTAGACACATTGTTGCTACCGCCACCCCCCACCCCCAACCCTACACTATTGCTGATACGAATTTCACAAAAGAATAAAACTGAAATGAGATACATGAAAAAAAAAAAA

**C**

ventricosus_f5e GTCAGACTGCCAGGATATATAAACCTCAGCCTCCACTCCTGGCCAACCGACACGCCTCCA

14357X3.C.furvu ------------------------------------------------------------

ventricosus_f5e GCTCCTCTCCCTCTCTCTCTCCACCCCTGACTCCATCGCTCTCCTTCCAGAAGCTCTCTT

14357X3.C.furvu ------------CACTCTCTGTCTCCCTGATTGC-------------------------T

* ****** . .******.* * *

ventricosus_f5e GGCATCAGCCAGGGAAACACTCTTGGGTCAATATCTGGCTCTCTCTCAACGCCCGGCTGC

14357X3.C.furvu GCCTTCAGTC--------------------------GACCCGCCCTCGTCGCCGCGCAG-

* * ****.* *.*.* *.***. **** ** *

ventricosus_f5e GTTTCTTCTCGAAAAGCTAACAGCCTTGTTGCTATCATAGGTCCGCGGAGAAGACGGTAG

14357X3.C.furvu ---ACCTGGTAAGAAGTGAAGAACCTT--TATCAT------------GGGGAAACTGACA

*.* ..*.***. ** *.**** *...** *.*.*.** * .

ventricosus_f5e GCACTTTTCATTGACGTTCTTGACAGCCTTAATCAACGGGTCATCCGTACAACAGAGGTC

14357X3.C.furvu ATACTGTTTCTTGTTGCTGCTG--------------------------------------

..*** **. *** .*.* .**

ventricosus_f5e TCGAGGGGCGAGGCTCTTTGGTTGGAGAACAAGTAGTAACAGGACGTGCGGATCAGAGGC

14357X3.C.furvu ------------------------------------------------------------

ventricosus_f5e GAAAGTTTGGGACGCAGTTGTCGCGGGACGCACCCCTGCTGGAAGAATTGGACGACCTGA

14357X3.C.furvu ------------CACTGTTGTCG-------------------------------------

*.* *******

ventricosus_f5e CGAGACAGTCCGTGAGTCATCTTCCCAAGGTCAGACACCGGCTCATCATGGTCCGAGCTG

14357X3.C.furvu ----------------------ATCCAGG----------------TCATGGTTCAAGGT-

.***.* *******.*.** *

ventricosus_f5e CTCTCTCCTTGCTGCTGCTGGCTACACTCGCCGTTCTCGTCATCGCGAATGAGCGAGCGG

14357X3.C.furvu --------------------------------------GACGGAGCGCATGAGCGAGCGA

* *. *** ***********.

ventricosus_f5e AAGCGGAAGAGCCACAGCATCACCGCGCGAAGCGACAGGACGACATGGCGGCGGTGGACG

14357X3.C.furvu AAGCGGAAGAGCCACAGCATCACCACGCGAAGCGACAGGACGGCA---------CGGACG

************************.*****************.** .*****

ventricosus_f5e ACTACCCTCTGGATGACGTGGACATGATGCAGCGTATTTTCCGCACCCCTCTCAAACGTC

14357X3.C.furvu GCTACCCTGTGGATGACGTGGACATGATGCAGCGTATTTTCCGCAACCCTCTCAAACGTC

.******* ************************************ **************

ventricosus_f5e AGTGGTGTCGGCCTGGCATGTCCTTCAACCCCGTGCTGGGCACCTGCACGCTGTCTCTGG

14357X3.C.furvu AGTGGTGTCGGGTTGGCTATGCCTACAACCCCGTGCTGGGCAGGTGCACGATATCTCTG-

*********** .**** *** ***************** ****** *.******

ventricosus_f5e CGGCGCTGCGAGGGCGAGGCA--GAAGTTTCCGTGGCGTCTGAGAGCCAGCC-GAGGGCG

14357X3.C.furvu -------TCGAGGATCAAGTATCCTGGGTTATATGAAATATATGAAGAAACCAGAAGGAG

*****.. *.*.* .* ** ..**. .* *. **. *.** **.** *

ventricosus_f5e CCAGTGATGACCCATACATGCTTGGTCCTGCCGACCGCTCTTGCCCAGTCCACTGTGCTT

14357X3.C.furvu CCAGTGATGACCCATACATGCTTGGTCCTGCCGACCACTCTT------------------

************************************.*****

ventricosus_f5e GTTTTGTCATTCGGTCACTAGAACCTGTCACGTTTTGGTTTACTTCCCTCCTCCCCCCCC

14357X3.C.furvu ------------------------------------------------------------

ventricosus_f5e CCCCTTCATCCTAGTCCCCTTCCACGCCCACCTCACCTACCACCTCTCCCACAAACCCCC

14357X3.C.furvu ------------------------------------------------------------

ventricosus_f5e CATTGATTTCGTCCTAGTGTATGTTCAGTTGTCATCAATGGGTTAAAGCCACGTCAACCT

14357X3.C.furvu ------------------------------------------------------------

ventricosus_f5e GAGTTGTAGTCACGAAGGTACAGTCACCCATGTGTTAAAGCAACGTGAATCCTTAGTTCA

14357X3.C.furvu ------------------------------------------------------------

ventricosus_f5e GTTCACGGAGGTACAGTCACTAATGGGTCAACGCGACCTGAATCCTGAATCTGTTCACAA

14357X3.C.furvu ------------------------------------------------------------

ventricosus_f5e AGATACGGCTATCAATGCGTTAAAGAAAATAAACCCTGAGACAGTCACATGAGTGTTTTA

14357X3.C.furvu ------------------------------------------------------------

ventricosus_f5e GCAATATGAATCCTTAGTTCTGCTCACGAAGGTTTCAGCAGTCAATGCGTTAAAAGCAAT

14357X3.C.furvu ------------------------------------------------------------

ventricosus_f5e GTGAATTCTTACTCCTTTTCTCGATGTTACAGTCATCAATGTGTTACAGCAAGATAACCC

14357X3.C.furvu ------------------------------------------------------------

ventricosus_f5e TAGTCCTGTTCACGATGTTACAGTCATCAATGTGTTACACAAGATAACCCTTTGATCTGT

14357X3.C.furvu ------------------------------------------------------------

ventricosus_f5e TCACGATGTTACAGTCATCAATGTGTTAAAGAAATGTAAAGCTTAGTCAGGTTCAAAATG

14357X3.C.furvu ------------------------------------------------------------

ventricosus_f5e ATAGGAGTCTTTAACATGTCAAAGTAGAACAAGTTCTGACCTATGTTCAGGACAACTAGG

14357X3.C.furvu ------------------------------------------------------------

ventricosus_f5e TCATTGAAGTGTCAAAACAAAATACAACCCAGGCTTTCTTCAAAATAACCTGCCCACTAA

14357X3.C.furvu ------------------------------------------------------------

ventricosus_f5e GGTTTCAAAGCAAACTAAGCTATTAGCTCTATTTACTTCGCTCTCTTCAACCTCATGAAT

14357X3.C.furvu ------------------------------------------------------------

ventricosus_f5e TACATCCACGAGTTAAAGCTCAAATAAACGTTCGGCTTTCTTACAGATAGTATTTCGGCT

14357X3.C.furvu --------------------------AACTTTCGGCTTTCTTACAGATAGTATTTCGGCT

*** ******************************

ventricosus_f5e TGATATGACTTCACCAGTAGACACAATGTTGCCACTCCCCCCCCCCCCCTCCAACCCCCC

14357X3.C.furvu TGATATGACTTTACCATTAGACACATTGTTGCTACCGCC--------------ACCCCCC

***********.**** ******** ******.**. ** *******

ventricosus_f5e ACCCCCACCCCTACACTATTGCTGATACGAATTTCACAAAGGAATAAAACTGAAATGAGA

14357X3.C.furvu ACCCCCAACCCTACACTATTGCTGATACGAATTTCACAAAAGAATAAAACTGAAATGAGA

******* ********************************.*******************

ventricosus_f5e TACAT-------------

14357X3.C.furvu TACATGAAAAAAAAAAAA

*****

**D**

Predicted based on boavistensis transcript

>SRR11807497.C.boavistensis.VG.TRINITY_DN58_c0_g1_i3 Entry:3024.conotoxin.O2 len:563 tpm:729.72

CATTCTTGTTCTTCTTTTCTATCTGCCTACCTCTCTCCCCGTGAGGAGGGTGAAGGTAAGCGACAGACGGTATAAATATTGGGGGAAAGCCGTCTCTCAAGCCATCTACACTCCGCCTGCTGACCCGTCGTCTACACTCTCTGTCTCCCTGATTGCTGCCTTCAGTCGACCCGCCCTCATCGCAGCGCAGACTTGGTAAGAAGTGAAGAACCTTTATCATGGAGAAACTGACAATACTGTTTCTTGTTGCTGCTGTACTGTTGTCGACCCAGGTCATGGTTCAAGGTGACGGAGATCAACCTGCAGATCGGGATGCAGTGCCAAGAGGCGGTACCTCATCTGGAACAAGTGGAAAGCTCATGAAAGCTCTACGTCAGTCTGATTGTCCATGGAACCCTTGGTGTGGCTGATCAGAATCAACGACTGCTATGACAGCCGACAGTGCCGCACTCGAATTACCCTACAGTGGTTTGATTGATCCAGAACAATGATGTTTTGAAAACCGTATTCGTTGCCGCGGCCATTTTTGTCGTGTTGTAATTAAATAAAGCGATGACAGTCTC

**E**

ventricosus_f5t TCTTGTTCTTCTTTTCTATCTGCCCACCTCTCTCCCCGTGAGGAGGGTGAAGGTTAGTGA

14357X3.C.furvu ------------------------------------------------------------

ventricosus_f5t CAGACGGTATAAATATTGGGGGAAAGCCGTCTCTCAAGCCATCTACACTCCGCCTGCTGA

14357X3.C.furvu ------------------------------------------------------------

ventricosus_f5t CCCGTCGTCTACACTCTCTGTCTCCCTGATTGCTGCCTTCAGTCGACCCGCCCTCATCGC

14357X3.C.furvu -----------CACTCTCTGTCTCCCTGATTGCTGCCTTCAGTCGACCCGCCCTCGTCGC

********************************************.****

ventricosus_f5t AGCGCAGACTTGGTAAGAAGTGAAGAACTGTTATCATGGAGAAACTGACAATACTGTTTC

14357X3.C.furvu CGCGCAGACCTGGTAAGAAGTGAAGAACCTTTATCATGGGGAAACTGACAATACTGTTTC

********.******************. *********.********************

ventricosus_f5t TTGTTGCTGCTGTACTGTTGTCGACCCTGGTCATGGTTCAAGGTGACGGAG---ATCAAC

14357X3.C.furvu TTGTTGCTGCTGCACTGTTGTCGATCCAGGTCATGGTTCAAGGTGACGGAGCGCATGAGC

************.***********.** *********************** ** *.*

ventricosus_f5t CTGCA-----GAACGTGATGCAGTGCCAAGAGACGATAACTCAGG-TGGAACGAGTGGAA

14357X3.C.furvu GAGCGAAAGCGGAAGAGCCACAGCATCACCACGCGAAGCGACAGGACGGCACGGACGGCT

**. *.* * * ..***...** * .*** . **** .** ***...**

ventricosus_f5t AGTTCATGAATG------------------------------------------------

14357X3.C.furvu ACCCTGTGGATGACGTGGACATGATGCAGCGTATTTTCCGCAACCCTCTCAAACGTCAGT

* ....**.***

ventricosus_f5t -------------------------CTCTACGTGGGGATTGTCCGTGGAATCCTTGGTGT

14357X3.C.furvu GGTGTCGGGTTGGCTATGCCTACAACCCCGTGCTGGGCAGGTGCACGATATCTCTGTCGA

*.*...*. *** ** *..*. ***..** .*

ventricosus_f5t GGCTGA------TCGGAATCTACGACTGCTATGA------------CAGCCGACAGTGCC

14357X3.C.furvu GGATCAAGTATCCTGGGTTATATGAAATATATGAAGAAACCAGAAGGAGCCAGTGATGAC

** * * ..**. * **.** ***** ****.....** *

ventricosus_f5t GCA-------------------------------CTCGAATTACCCTACAGTGGTTTGAT

14357X3.C.furvu CCATACATGCTTGGTCCTGCCGACCACTCTTAACTTTCGGCTTTCTTACAGATAGTATTT

** .*. ...* .*.***** . * *

ventricosus_f5t TGATCCAGAACAATGATGTTTTGAAAACCGTATTCGTTGCCGCGGC--------------

14357X3.C.furvu CGGCTTGATATGACTTTACCATTAGACACATTGTTGCTACCGCCACCCCCCACCCCCAAC

.*...... *..*. *... * *.* *.* *.*.*.**** .*

ventricosus_f5t ----CATTTTTGTCG-----------------------TGTTGTAATTAAATAAAGCGAT

14357X3.C.furvu CCTACACTATTGCTGATACGAATTTCACAAAAGAATAAAACTGAAATGAGATACATGAAA

**.* ***..* ..** *** *.*** * .*

ventricosus_f5t GACAGTCTC

14357X3.C.furvu AAAAAAAAA

.* *.
