# Supplementary material for: A toxin-based approach to neuropeptide and peptide hormone discovery
Source: Front Mol Neurosci. 2023 Aug 31;16:1176662. doi: 10.3389/fnmol.2023.1176662 (PMC10501145; doi:10.3389/fnmol.2023.1176662)
Supplement: Supplementary file 14 [file Data_Sheet_14.docx]

Supplementary Material

A toxin-based approach to neuropeptide and peptide hormone discovery

**Thomas Lund Koch^1,2*^, Joshua P. Torres^1^, Robert P. Baskin^3,4^, Paula Flórez Salcedo^5^, Kevin Chase^3^, Baldomero M. Olivera^3^, and Helena Safavi-Hemami^1,2,3*^**

^1^ Department of Biomedical Sciences, University of Copenhagen, Copenhagen-N, Denmark

^2^ Department of Biochemistry, University of Utah, Salt Lake City, UT, USA

^3^ School of Biological Sciences, University of Utah, Salt Lake City, UT, USA

^4^ The Ohio State University College of Medicine, Columbus, OH, USA

^5^ Department of Neurobiology, University of Utah, Salt Lake City, UT, USA

*** Correspondence:**Thomas Lund Koch* thomas.koch@biochem.utah.edu

Helena Safavi-Hemami* helena.safavi@utah.edu

## Supplementary Figures


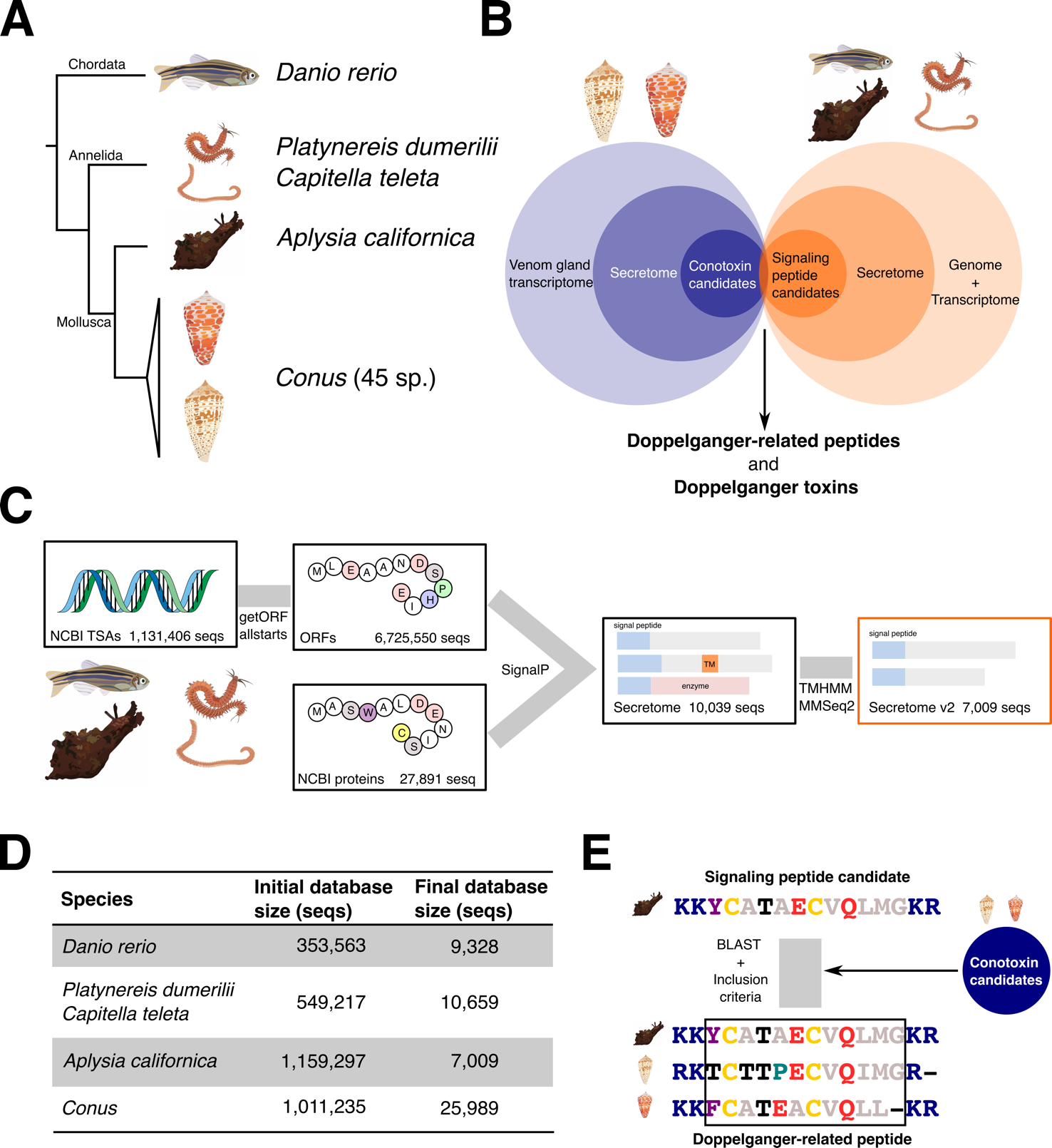


**Supplementary Figure 1.** Methodology of toxin-based approach to signaling peptide discovery. (**A**) Model species used in this study representing cone snail prey: *Danio rerio* (zebrafish), the two annelids *Platynereis dumerilii* and *Capitella teleta*, and the mollusk *Aplysia californica* (Californian sea hare) (**B**) The toxin-based approach to signaling peptide discovery is based on finding signaling peptide candidates that share homology with putative conotoxins. (**C**) Workflow for building databases of putative signaling peptide candidates from prey species: predicted proteins and transcriptome assemblies were downloaded and filtered using SignalP. Sequences containing transmembrane domains and those with similarity to known enzymes were removed. A similar approach was used to extract conotoxin candidates from 45 different species of cone snails. (**D**) Resulting database size: 9,328 sequences for *D. rerio*, 7,009 sequences for *A. californica,* and 10,659 sequences for *C. teleta* and *P. dumerilii*. (**E**) Putative prey signaling peptides were blasted against the conotoxin library and retained based on criteria listed under Materials and Methods.

**
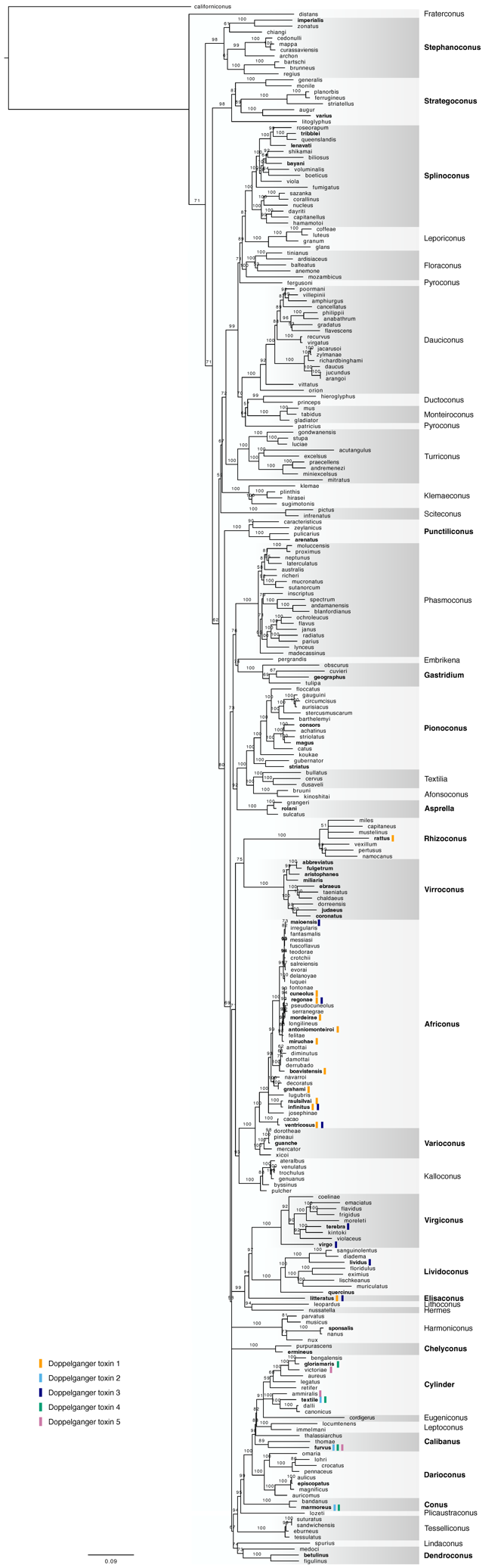
**

**Supplementary Figure 2.** Maximum likelihood tree based on concatenation of COI, 16S, and 12S genes from Conus. The bolded species were included in the venom gland transcriptomes analyzed in this study. The presence and absence of identified DREPs are marked next to the species name. The tree is rooted with *Californiconus californicus*genes.

**
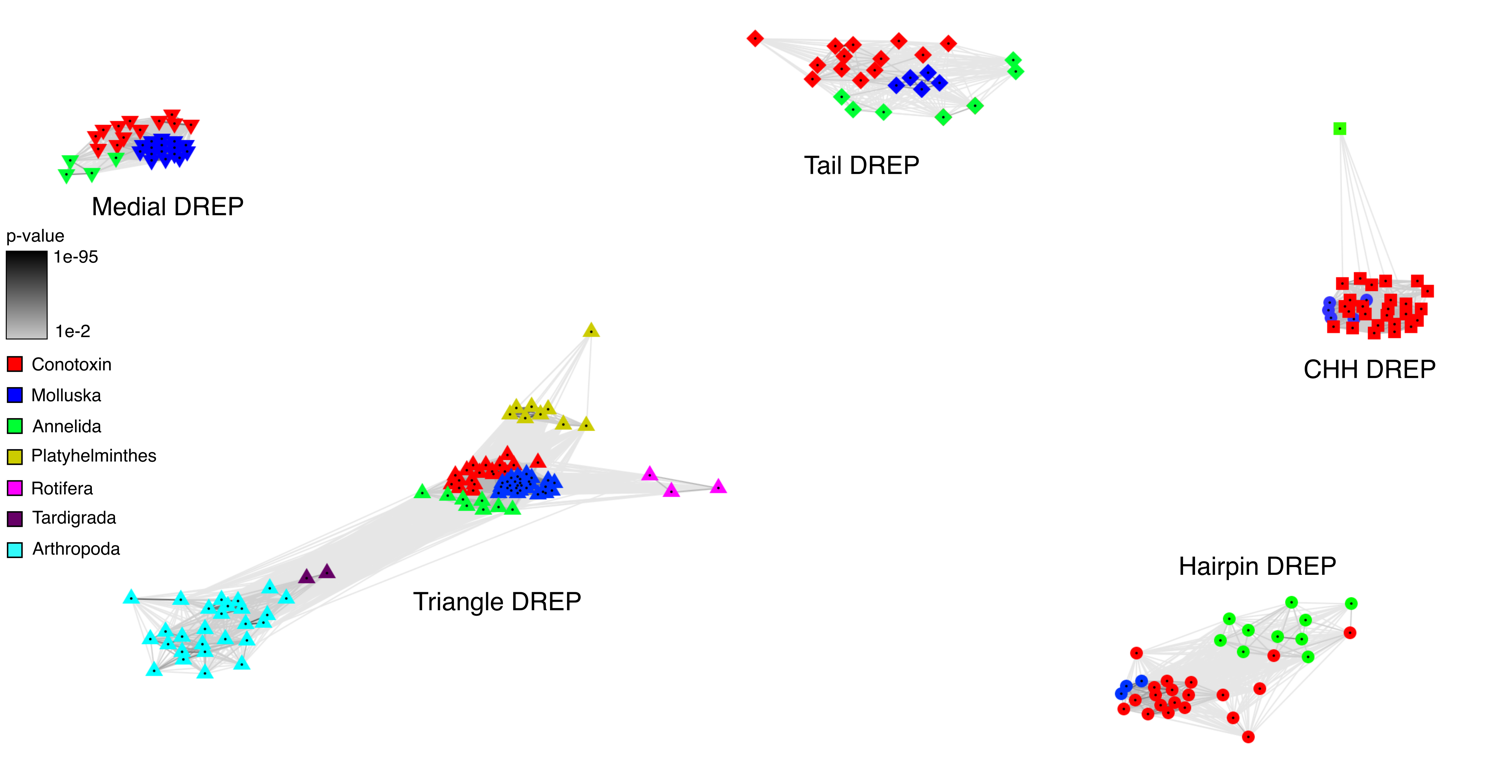
**

**Supplementary Figure 3.** BLOSUM62 map of doppelganger toxins and DREPs. The nodes represent individual precursor sequences, and the edges correspond to the BLASTp p-values linking the ends.

**
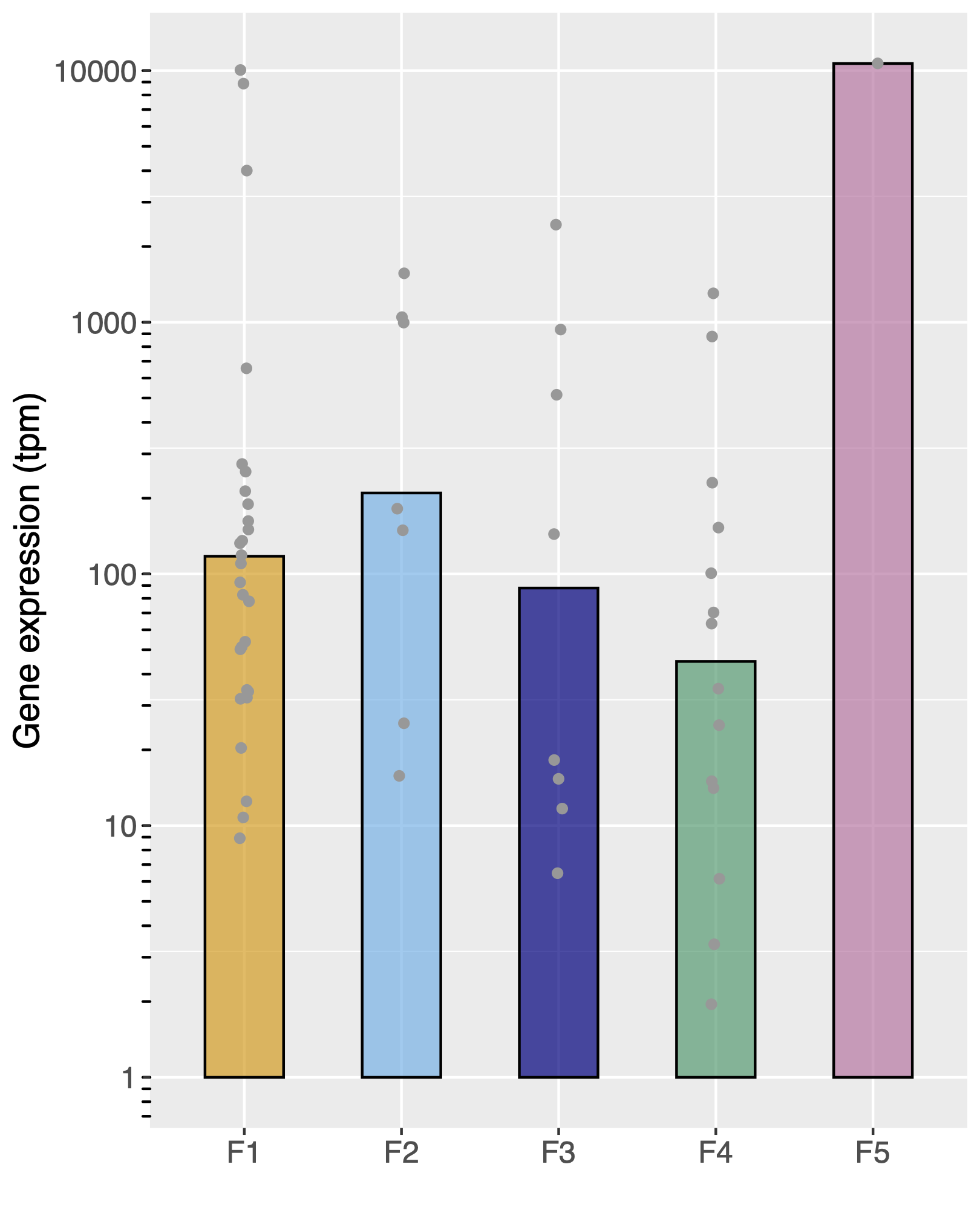
Supplementary Figure 4.** Mean doppelganger toxin gene expression in cone snail venom glands. Dots represent individual data points.

**
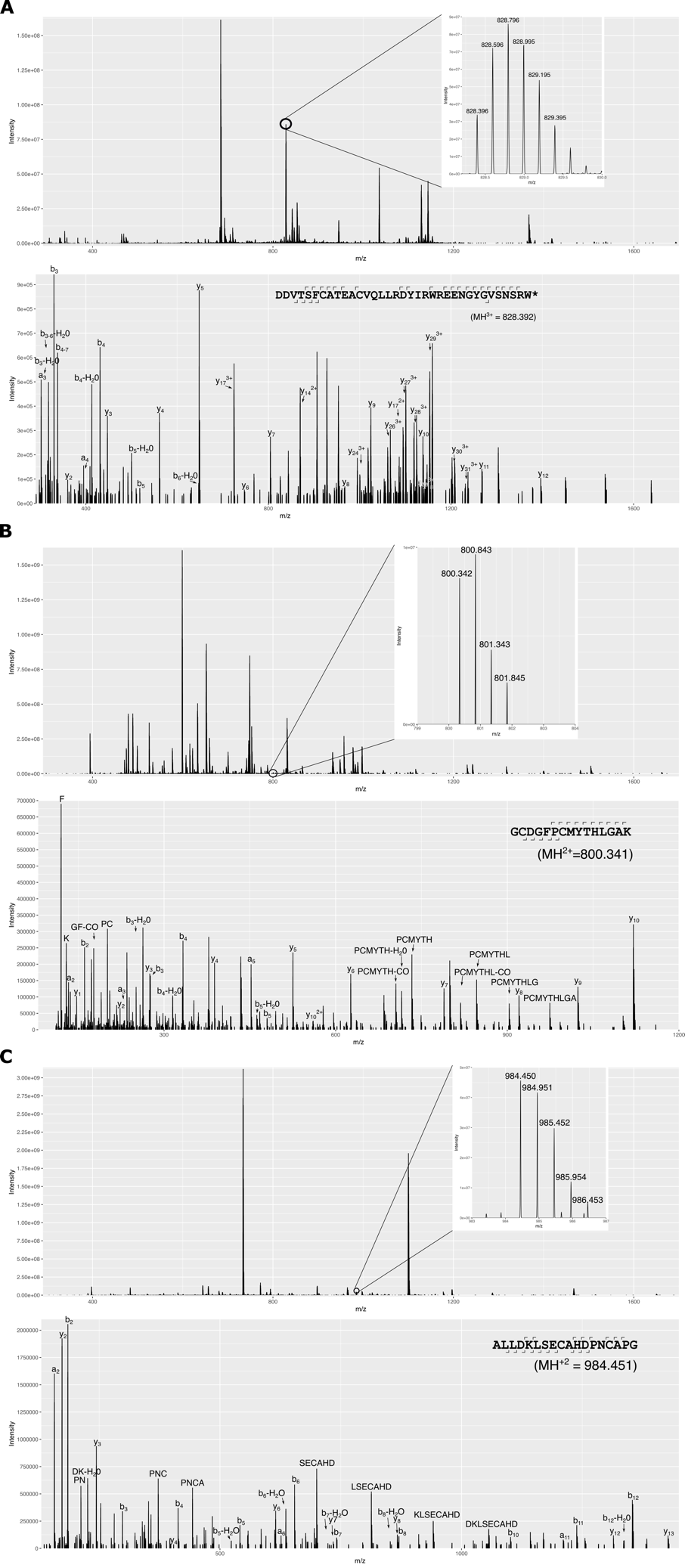
**

**Supplementary Figure 5.** Mass spectrometry of doppelganger toxins. MS (upper) and MS/MS (lower) spectra of Tail and Medial doppelganger toxins. (A) *Conus terebra*Tail doppelganger toxin is C-terminally amidated, (B) *Conus textile*Medial doppelganger toxin 1, (C) *Conus marmoreus*Medial doppelganger toxin 2.

**
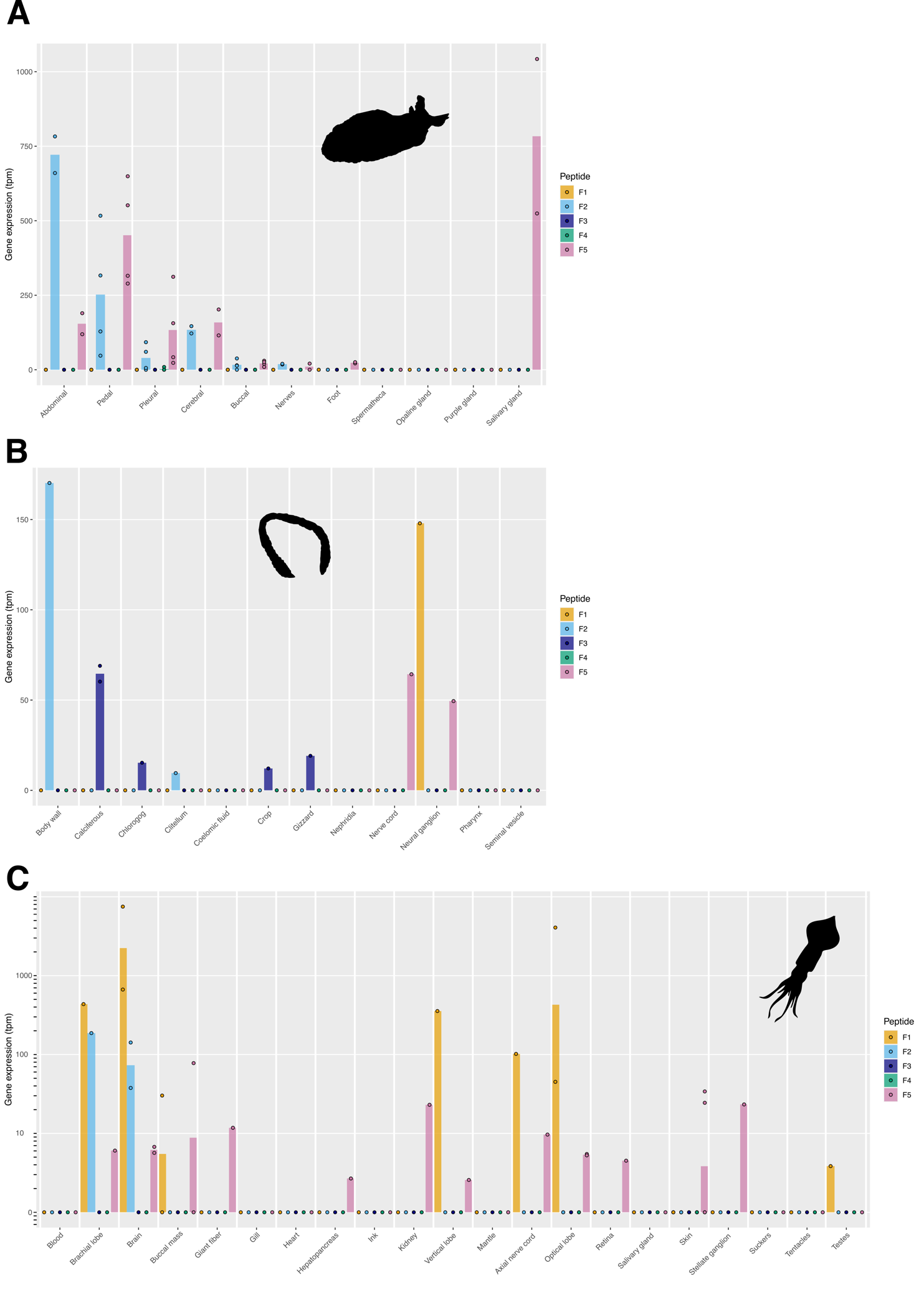
Supplementary Figure 6.** DREP transcript expression. Mean (A) Aplysia-DREP, (B) Lumbricus-DREP, and (C) Doryteuthis-DREP transcript expression in different tissues.

**
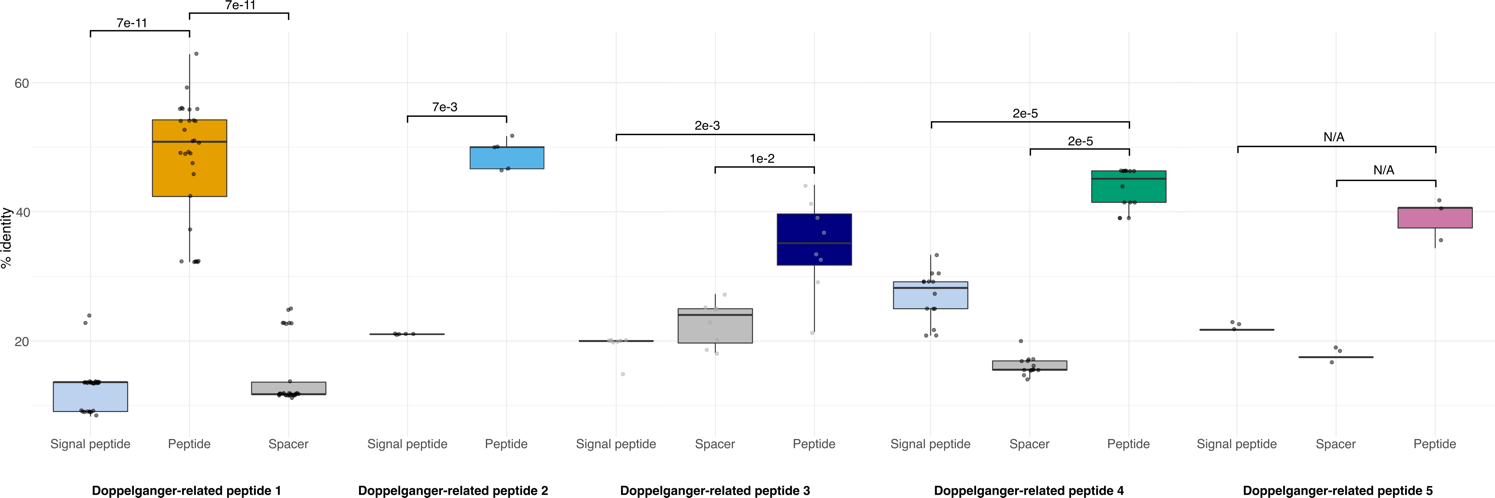
**

**Supplementary Figure 7.** Amino acid identity of doppelganger toxins to prey. Amino acid identity of doppelganger toxins to prey DREP separated into the signal sequence, peptide/toxin region, and where applicable spacer region(s). Significance is calculated by Wilcoxon rank-sum test and p-values compared to the peptide/toxin regions are shown.

Evolutionary trace analyses. Evolutionary trace analyses show different conservation (rate4site) scores in the toxin/peptide regions compared to the signal sequence and spacer region(s). (Left) Position-specific rate4site scores for doppelganger toxin and DREPs. (Right) Wilcoxon rank-sum test shows differences between the toxin region compared to the signal sequence and spacer region. The signal peptide is depicted in light blue, processing sites are in red, and cysteines in yellow. The peptide and toxin regions are shown above the graphs. Spacer regions are defined as the non-signal sequence/peptide/processing site regions.

**
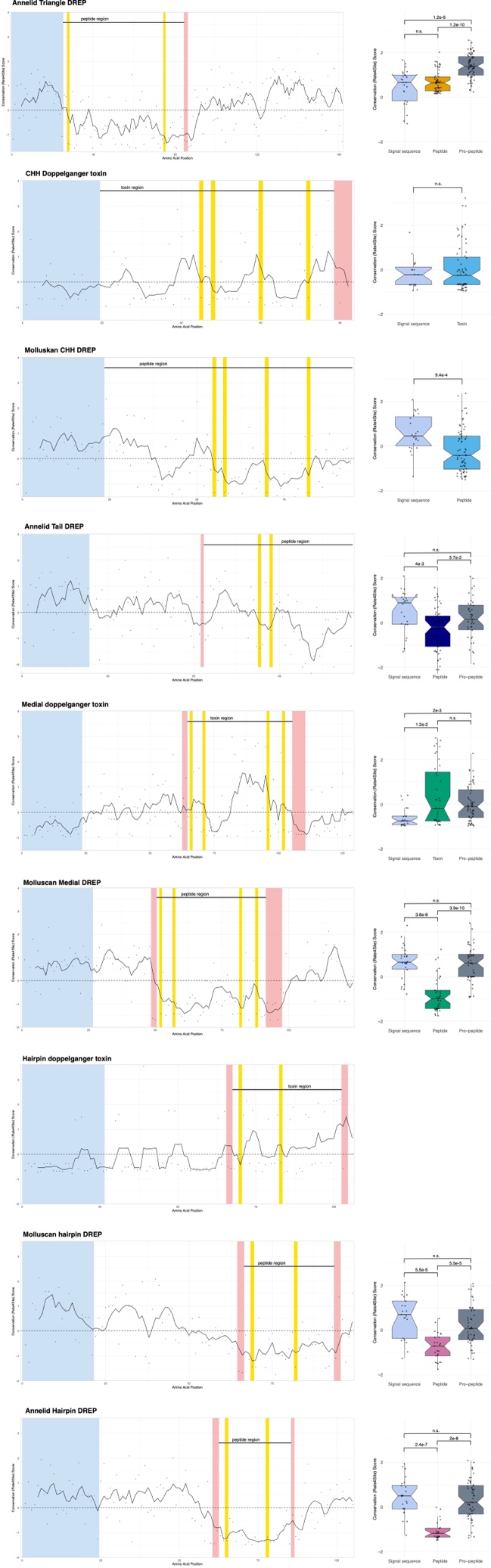
**

**Supplementary Figure 8.** Wilcoxon rank-sum test of rate4site scores of exon1 and exon2 of Triangle doppelganger toxin.
